# Fibrinogen triggers perivascular fibroblast activation in a mouse model of cortical ischemic stroke

**DOI:** 10.1016/j.isci.2025.113834

**Published:** 2025-10-24

**Authors:** Jose C. Martínez Santamaría, Corey Fehlberg, Pasquale Conforti, Jan N. Ness, Francesca Garafulic Justiniano, Pedro Manzitti, Felicitas Bucher, Jae K. Lee, Christian Schachtrup

**Affiliations:** 1 Institute of Anatomy and Cell Biology, Faculty of Medicine, University of Freiburg, 79104 Freiburg, Germany; 2 Faculty of Biology, University of Freiburg, 79104 Freiburg, Germany; 3 Miami Project to Cure Paralysis, Department of Neurological Surgery, University of Miami School of Medicine, Miami, FL 33136, USA; 4 Eye Center, Medical Center, Faculty of Medicine, University of Freiburg, Freiburg, Germany; 5 Institute of Pharmaceutical Sciences, University of Freiburg, Freiburg, Germany; 6 Center for Basics in NeuroModulation (NeuroModulBasics), Faculty of Medicine, University of Freiburg, Freiburg, Germany

**Keywords:** Physiology, Neuroscience, cell biology

## Abstract

Fibrotic scar formation caused by stromal cells is often associated with chronic, non-healing pathology impeding repair of the central nervous system (CNS). Perivascular fibroblasts (PVFs) in the perivascular space are activated, express and deposit excess collagen I (Col I), and form a fibrotic scar following CNS disease. Here we show that blood-derived fibrinogen deposition in the perivascular space following photothrombosis, a mouse model for ischemic stroke, initially induces PVF activation. Pharmacological fibrinogen depletion reduces PVF activation and migration away from blood vessels to build up the fibrotic scar. Fibrinogen-induced beta1 integrin signaling in PVF regulates Col I expression. Single-cell RNA sequencing and genetic approches revealed a contribution of fibrinogen-induced myeloid cells to PVF activation. Fibrinogen depletion abrogates PVF-astrocyte signaling and lesion border formation, promoting neuronal survival and plasticity. We propose that fibrinogen is a critical trigger for fibrotic scar formation, inhibiting neuronal regeneration after stroke.

## Highlights

• Cortical injury leads to immediate fibrinogen deposition in the perivascular space
• Fibrinogen induces PVF activation and Col I expression via β1 integrin signaling
• Fibrinogen-induced myeloid cell activation contributes to PVF activation
• Fibrinogen orchestrates lesion border formation, hindering neuronal regeneration

## Graphical abstract

**Figure:**
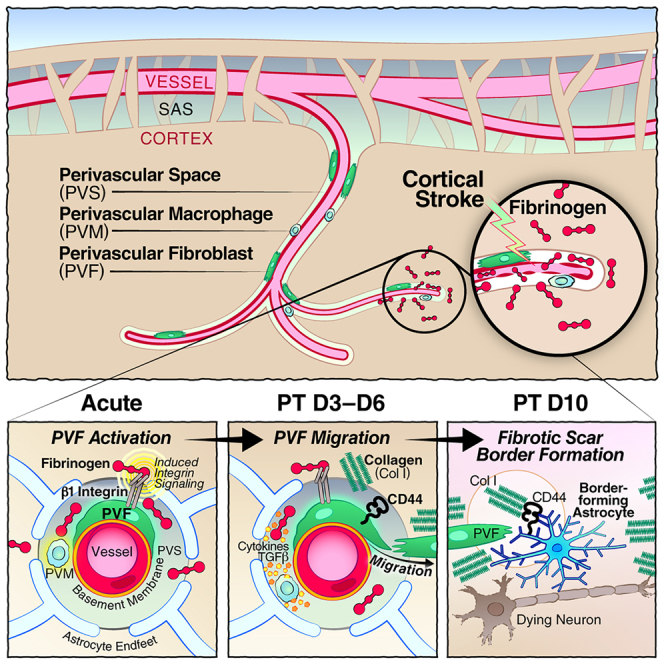

## Introduction

Fibrotic scar formation in the central nervous system (CNS) is driven by stromal cells depositing an excess of extracellular matrix (ECM) proteins (e.g., collagen I (Col I)) that is often associated with chronic, non-healing pathology that impedes CNS repair.^1^^,^^2^^,^^3^^,^^4^^,^^5^ Perivascular fibroblasts (PVFs) located in the perivascular space are loosely associated with larger blood vessels and express ECM structural components and its modifiers or receptors, suggesting a function in basement membrane protein production and contribution to cerebrovascular structures in rodents and humans in healthy conditions.^6^^,^^7^^,^^8^^,^^9^^,^^10^ Once activated after CNS disease (e.g., after stroke), PVF proliferate, migrate away from the vasculature and express and secrete an excess of Col I, contributing significantly to fibrotic scar formation.^11^^,^^12^^,^^13^^,^^14^

One of the earliest events in CNS pathologies, such as multiple sclerosis, Alzheimer’s disease, traumatic brain injury and stroke, is leakage of blood components into brain parenchyma^15^^,^^16^ at areas that correlate with scar formation.^17^ Fibrinogen (coagulation factor I) is a 340–kDa protein secreted by hepatocytes in the liver and present in the blood circulation at 3–5 mg/mL. Fibrinogen is cleaved by thrombin and, upon conversion to fibrin, serves as the major architectural protein component of blood clots.^16^^,^^18^ Beyond its function in blood clotting, fibrinogen acts as a multi-faceted signaling molecule that influences inflammatory, neurodegenerative and repair processes in the injured CNS.^19^ Fibrinogen mediates functions in the nervous system as a ligand for cell-specific receptors. In microglia, fibrinogen induces activation of Akt and Rho via the CD11b/CD18 integrin receptor (complement receptor 3).^20^^,^^21^ In astrocytes, fibrinogen as a carrier of latent TGF-β induces TGF-β receptor activation to regulate astrocyte activation and lesion border formation.^17^^,^^22^ Recent advancements in animal models and human tissue analysis implicate the blood-derived coagulation factor fibrinogen as a molecular link between vascular permeability and astrocyte activation and lesion border formation.^17^^,^^22^^,^^23^^,^^24^ However, the role of fibrinogen in PVF activation and fibrotic scar formation after stroke remains unknown.

In this study, we investigated the role of fibrinogen in the activation of PVF in mice subjected to photothrombotic ischemia (PT), a mouse model of cortical ischemic stroke. Here, we show that cortical PT-evoked fibrinogen deposition in the perivascular space precedes PVF activation. Pharmacological depletion of fibrinogen reduced PVF activation, migration and deposition of Col I via beta1 integrin signaling, reducing overall fibrotic scar formation. Single-cell RNA sequencing (scRNA-seq) and genetic approaches revealed a contribution of fibrinogen-induced myeloid cells to PVF activation. Finally, depletion of fibrinogen reduces PVF-astrocyte interactions and lesion border formation and increased neuronal survival and plasticity after PT. Consequently, we propose that fibrinogen is an initial regulator of PVF activation in the perivascular space via integrin signaling after stroke.

## Results

### Fibrinogen deposition precedes PVF activation in the perivascular space after cortical injury

**Figure 1:**
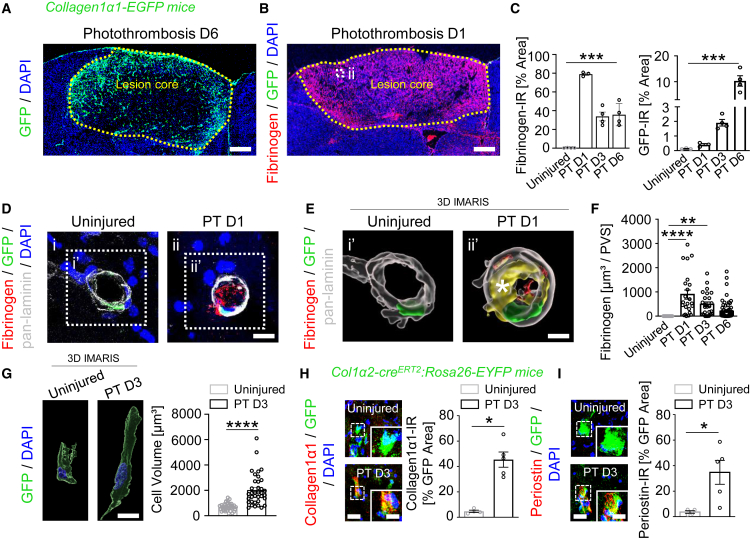
Fibrinogen deposition precedes PVF activation in the perivascular space after cortical injury (A) Representative image of GFP (green) in *collagen1α1-EGFP* mice in the lesion core 6 days after PT (*n* = 4 mice). Scale bar, 400 μm. (B) Representative image of fibrinogen (red) and GFP (green) in the lesion core 1 day after PT (*n* = 3 mice). White dashed box (ii) indicate the enlargement of a vessel area presented in Figure 1D. Scale bar, 400 μm. (C) Quantification of fibrinogen- (left) and GFP- (right) immunoreactivity (IR) in the lesion core per area (*n* = 4 mice, uninjured, PT D3, PT D6; *n* = 3 mice, PT D1). ∗∗∗*p* < 0.001 by one-way ANOVA. (D) Representative images of fibrinogen (red), GFP (green) and pan-laminin (gray) in the vasculature of the lesion core 1 day after PT compared to uninjured brain. White dashed boxes (i’, ii’) indicate enlargement of vessel areas presented in Figure 1E. Scale bar, 10 μm. (E) 3D reconstruction of the cortical vasculature 1 day after PT showing fibrinogen deposition (white star) in the perivascular space (yellow), compared to the uninjured brain. Scale bar, 5 μm. (F) Quantification of fibrinogen and pan-laminin colocalization in the perivascular space (*n* = 3 mice; 18 vessel areas, uninjured; 23 vessel areas, PT D1; 25 vessel areas, PT D3; 71 vessel areas, PT D6). ∗∗*p* < 0.01 by one-way ANOVA; ∗∗∗∗*p* < 0.0001 by one-way ANOVA. (G) 3D reconstruction of GFP+ cells in the perivascular space in the lesion core of mice 3 days after PT, compared to uninjured mice. Scale bar, 10 μm. Quantification of GFP+ cell volume (*n* = 3 mice, 36 cells). ∗∗∗∗*p* < 0.0001 by Mann-Whitney U test. (H) Representative images of collagen1*α*1 (red) in combination with GFP (green) in the perivascular space in the lesion core of *collagen1α2-cre*^*ERT2*^*:Rosa26-EYFP* mice 3 days after PT, compared to uninjured mice. Scale bars, 10 μm (overviews), 5 μm (magnifications). Quantification of collagen1α1–IR per GFP+ area (*n* = 4 mice, uninjured; *n* = 5 mice, PT D3). ∗*p* < 0.05 by Mann-Whitney U test. (I) Representative images of periostin (red) in combination with GFP (green) in the perivascular space in the lesion core of *collagen1α2-cre*^*ERT2*^*:Rosa26-EYFP* mice 3 days after PT, compared to uninjured mice. Scale bars, 10 μm (overviews), 5 μm (magnifications). Quantification of periostin-IR per GFP+ area (*n* = 4 mice, uninjured; *n* = 5 mice, PT D3). ∗*p* < 0.05 by Mann-Whitney U test. All data are shown as mean ± s.e.m. See also Figures S1 and S2.

To investigate the role of fibrinogen on PVFs and fibrotic scar formation, we first examined the time course of fibrinogen deposition and PVF activation in a mouse model of cortical ischemic stroke (PT) using mouse lines genetically targeting CNS fibroblasts (*collagen1α1-EGFP* and *collagen1α2-Cre*^*ERT2*^^7^^,^^11^^,^^12^^,^^25^). After PT, we detected immediate and massive fibrinogen deposition in the cortical lesion area at day 1 after PT and before the increase of GFP+ cells in *collagen1α1-EGFP* transgenic mice forming the fibrotic scar at days 3 and 6 after PT (Figures 1A–1C, S1A–S1D, and S2A). PVFs reside in the perivascular space of large-diameter arterioles and venules, but not the capillaries.^2^^,^^6^^,^^7^^,^^9^^,^^12^ Morphometric analysis and 3–D reconstruction of blood vessels in the cortical lesion area revealed that fibrinogen colocalized with GFP+ cells in the perivascular space at day 1 and day 3 after PT, whereas fibrinogen was absent in healthy mice (Figures 1D–1F). Of note, individual GFP+ PVFs revealed a morphological change and an increased cell volume 3 days after PT (Figure 1G), indicating their early activation in the perivascular space. In accordance, protein expression levels of Col I and periostin, both activated fibroblast-secreted ECM proteins,^11^^,^^26^ were increased in GFP+ PVFs in the first days after PT in the tamoxifen-inducible *collagen1α2-Cre*^*ERT2*^*:Rosa26-EYFP* and *collagen1α1-EGFP* fibroblast-targeting mouse lines (Figures 1H, 1I, S2B, and S2C). These observations suggest that fibrinogen deposition in the perivascular space is an early inducer of PVF activation.

### Fibrinogen deficiency reduces PVF activation

**Figure 2:**
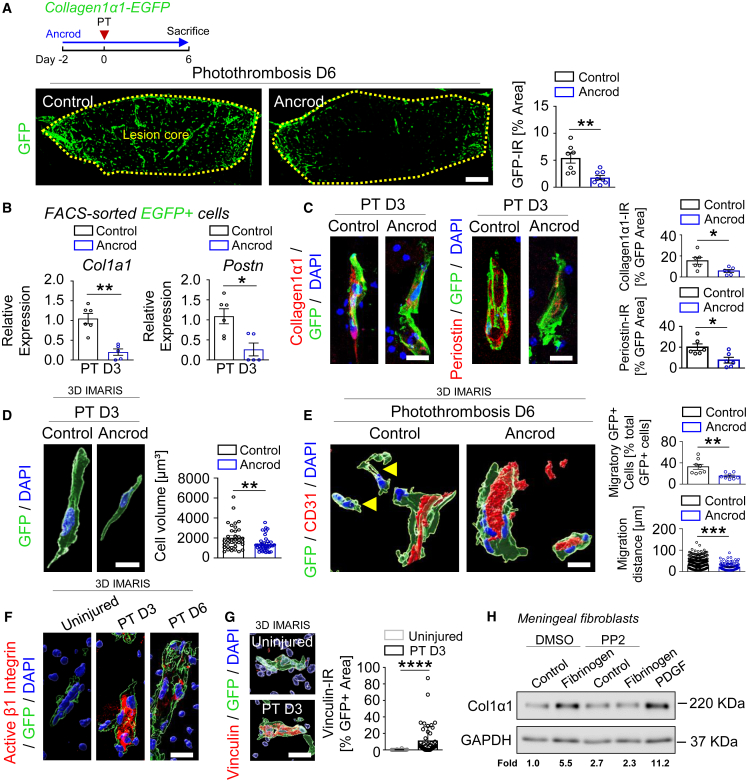
Fibrinogen deficiency reduces PVF activation after PT (A) Experimental setup for photothrombosis (PT) on fibrinogen-depleted (ancrod) and NaCl-treated (control) *collagen1α1-EGFP* mice. Representative images of GFP (green) in the lesion core of fibrinogen-depleted and control mice 6 days after PT. Scale bar, 400 μm. Quantification of GFP-immunoreactivity (IR) in the lesion core per area (*n* = 7 mice, control; *n* = 8 mice, ancrod). ∗∗*p* < 0.01 by Student’s *t* test. (B) Expression of *collagen1α1* and *periostin* mRNA in FACS-isolated GFP+ cells of fibrinogen-depleted and control *collagen1α1-EGFP* mice 3 days after PT determined by qPCR and normalized to GAPDH (*n* = 6 mice, control; *n* = 5 mice, ancrod). ∗*p* < 0.05, ∗∗*p* < 0.01 by Mann-Whitney U test. (C) Representative images of collagen1*α*1 (red, left) and periostin (red, right) in combination with GFP (green) in the perivascular space in the lesion core of fibrinogen-depleted and control mice 3 days after PT. Scale bar, 20 μm. Quantification of Collagen1α1- and periostin-IR per GFP+ area (*n* = 6 mice). ∗*p* < 0.05 by Mann-Whitney U test. (D) 3D reconstruction of GFP+ cells in the perivascular space in the lesion core of fibrinogen-depleted and control mice 3 days after PT. Scale bar, 10 μm. Quantification of GFP+ cell volume (*n* = 3 mice; 36 cells, control; 39 cells, ancrod). ∗∗*p* < 0.01 by Mann-Whitney U test. (E) 3D reconstruction of GFP (green) and CD31 (red) in the lesion core of fibrinogen-depleted (ancrod) and control mice 6 days after PT, compared to uninjured mice. Yellow arrowheads indicate migratory PVF. Scale bar, 10 μm. Quantification of migratory PVF (*n* = 8 mice). ∗∗*p* < 0.01, by Student’s *t* test. Quantification of PVF migration distance away from vasculature (*n* = 8 mice; 504 cells, control; 125 cells, ancrod). ∗∗∗*p* < 0.001 by Mann-Whitney U test. (F) 3D reconstruction of active β1 integrin (red), and GFP (green) in the lesion core 3 and 6 days after PT, compared to uninjured mice (*n* = 3 mice). Scale bar, 10 μm. (G) 3D reconstruction of vinculin (red), and GFP (green) in the lesion core 3 days after PT, compared to uninjured mice. Scale bar, 20 μm. Quantification of vinculin-IR in GFP+ area (*n* = 6 mice; 32 cells, control; 49 cells, ancrod). ∗∗∗∗*p* < 0.0001 by Mann-Whitney U test. (H) Representative western blot image of collagen1α1 expressed by meningeal fibroblasts pretreated for 1 h with PP2 and treated for 1 day with fibrinogen. PDGF served as a positive control (*n* = 4 biological replicates). All data are shown as mean ± s.e.m. See also Figures S3–S7.

To determine if fibrinogen is required for PVF activation and fibrotic scar formation after PT, we used systemic administration of the pharmacologic reagent ancrod to deplete fibrinogen before PT and for the duration of the experiment. Fibrinogen depletion resulted in a ∼65% fewer activated PVFs in the lesion core at day 6 after PT than in control mice (Figure 2A), while it did not change the overall lesion size at day 7 after PT (Figure S3A). Importantly, fibrinogen depletion revealed a repression of *collagen1α1* and *periostin* mRNA expression by FACS-sorted GFP+ PVFs at day 3 after PT, the time point when these cells are still located in the perivascular space, compared to vehicle-treated mice (Figure 2B). In line, fibrinogen depletion reduced Col I and periostin protein expression in GFP+ cells both by ∼62.5% at day 3 after PT and by ∼57% and ∼78% at day 6 after PT, respectively (Figures 2C and S3B). Furthermore, fibrinogen depletion decreased the PVF cell volume as determined by 3–D reconstruction at day 3 after PT (Figure 2D), indicating that fibrinogen regulates PVF activation already within the perivascular space. Of note, fibrinogen depletion significantly reduced Col I protein expression 7 days after laser-induced choroidal neovascularization (CNV), a mouse model of age-related macular degeneration (AMD),^27^ compared to the control group (Figures S4A–S4C), suggesting that fibrinogen not only regulates fibrosis and Col I expression in stroke, but also in other CNS diseases.

Next, we analyzed whether fibrinogen regulates PVF overt migration away from blood vessels to form a component of the fibrotic scar and express and secrete pathological matrix deposition. Fibrinogen alters the alpha smooth muscle actin (α-SMA) immunoreactivity *in vivo* and *in vitro* (Figures S5A and S5B), suggesting a potential transformation of PVF into migrating myofibroblast-like cells.^28^ PVF reside between the mural cell layer and astrocytic endfeet,^2^^,^^6^^,^^7^^,^^12^ and astrocyte endfeet coverage of blood vessels in the lesion core is disrupted at day 3 after PT (Figure S5C), the time point when PVFs initiate their overt migration away from blood vessels (Figure S5D). CD44, a cell-surface receptor sensor for ECM changes^29^ and important for fibroblast migration (Figure S6A), is induced in GFP+ PVFs 3 days after PT (Figure S6B) and regulated by fibrinogen *in vitro* (Figure S6C). Importantly, fibrinogen depletion reduced the migratory GFP+ cell number and their migration distance from the vasculature by ∼55% and ∼30%, respectively, 6 days after PT, compared to the control group (Figures 2E and S6D). This suggests that immediate fibrinogen deposition in the perivascular space regulates the initial PVF activation, their expression of cell-surface receptors for migration, and their overt migration away from the vasculature to the lesion core to build up the fibrotic scar after PT.

Fibrinogen acts as a provisional matrix inducing integrin and non-integrin receptor-mediated signaling, potentially leading to changes in PVF morphology and function.^16^ Fibroblasts sense the mechanical properties of the ECM by integrin and non-integrin mechanoreceptors,^30^ and given their positioning in the perivascular space, PVFs likely receive their activation cues via an altered perivascular space composition, such as fibrinogen deposition, leading to PVF activation. Indeed, immunolabeling for active β1 integrin (9EG7 antibody^31^) revealed its drastic appearance in PVFs at day 3 after PT (Figure 2F), coinciding with fibrinogen deposition and initial PVF activation. Active β1 integrin was barely detectable at day 6 after PT and in healthy mice (Figure 2F). In PVFs 3 days after PT, levels of vinculin, a mechanosensitive component of integrin adhesions,^32^ were greater than the base level expression in heathy mice (Figure 2G). A common mechanism that transmits integrin signaling is activation of Src family kinases (SFK).^33^ Neutralization of β1 integrin signaling by applying an SFK inhibitor resulted in a ∼60% decrease in fibrinogen-induced PVF Col 1 expression and secretion (Figures 2H and S6E), indicating that fibrinogen-induced β1 integrin-mediated mechanotransduction is regulating PVF activation and ECM secretion after PT. Of note, fibrinogen depletion by ancrod administration affected neither the cell number nor proliferation of GFP+ PVFs in the perivascular space at day 3 after PT, whereas PVF cell death was affected on a very low level (∼4% in control vs. ∼8% in ancrod) (Figures S7A–S7C). These data suggest that immediate fibrinogen deposition regulates β1 integrin signaling-mediated initial PVF activation in the perivascular space, and their overt migration away from the vasculature to build up the fibrotic scar after PT.

### Fibrinogen-induced myeloid cells contribute to PVF activation

**Figure 3:**
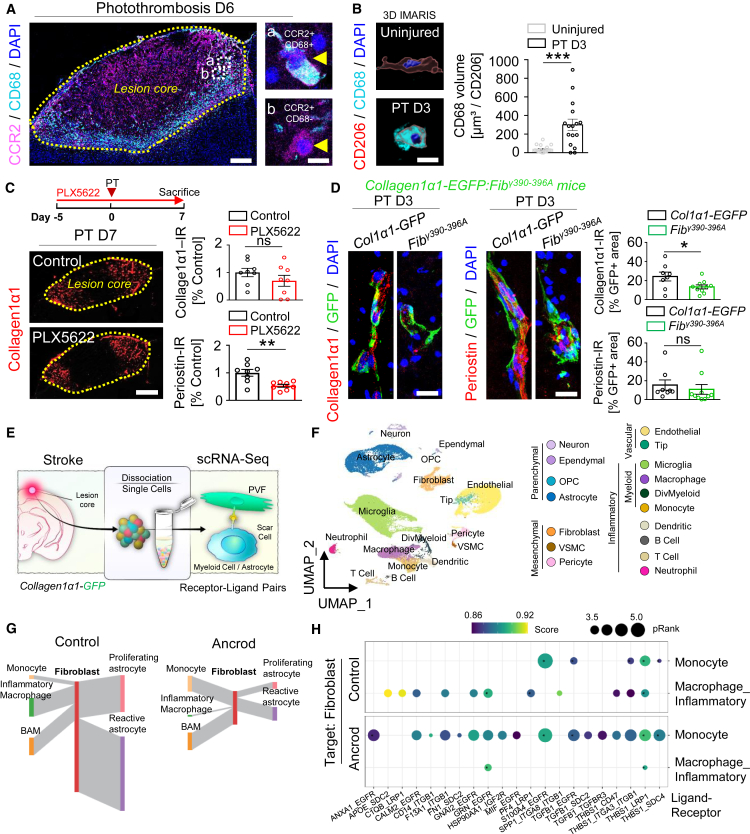
Fibrinogen-induced myeloid cells contribute to PVF activation after PT (A) Representative image of CCR2 (magenta) and CD68 (cyan) in the lesion core 6 days after PT (*n* = 1 mouse). White dashed boxes (a, b) indicate the enlargement of CCR2+ cells (right). Yellow arrowheads indicate a CCR2+CD68^+^ cell (right, top) and a CCR2+CD68^–^ cell (right, bottom). Scale bars, 400 μm (overview), 5 μm (magnifications). (B) 3D reconstruction of CD206 (red) and CD68 (cyan) in the perivascular space in the lesion core 3 days after PT, compared to uninjured mice. Scale bar, 5 μm. Quantification of CD68 volume in CD206^+^ perivascular macrophages (*n* = 2 mice; 19 cells, uninjured; 16 cells, PT D3). ∗∗∗*p* < 0.001 by Mann-Whitney U test. (C) Experimental setup for photothrombosis (PT) on myeloid cells-depleted (PLX5622)- and chow-fed (control) mice. Representative images of collagen1α1 (red) in the lesion core in myeloid cell-depleted and control mice 7 days after PT. Scale bar, 400 μm. Quantification of collagen1α1- and periostin-immunoreactivity (IR) in the lesion core per area normalized by the control group (*n* = 8 mice). ns, not significant, ∗∗*p* < 0.01 by Student’s *t* test. (D) Representative images of collagen1*α*1 (red, left) and periostin (red, right) in combination with GFP (green) in the perivascular space in the lesion core of *collagen1α1-EGFP:Fib*^*γ390–396A*^ mice, compared to *collagen1α1-EGFP* mice 3 days after PT. Scale bars, 20 μm. Quantification of collagen1α1- and periostin-IR per GFP+ area normalized by the control group (*n* = 8 mice, *collagen1α1-EGFP*; *n* = 10 mice, *collagen1α1-EGFP:Fib*^*γ390–396A*^). ns, not significant and ∗*p* < 0.05 by Student’s *t* test (top) and Mann-Whitney U test (bottom). (E) scRNA-seq of the lesion core after PT and receptor-ligand pair analysis of PVFs and other scar cells. (F) UMAP representation of individual cells from the lesion core in fibrinogen-depleted and control mice 6 days after PT by scRNA-seq. Each dot represents an individual cell. OPC, oligodendrocyte precursor cell. VSMC, vascular smooth muscle cell. (G) Sankey diagram depicting cell interactions between different cell populations in fibrinogen-depleted and control mice 6 days after PT. Thickness of gray lines represents number of interactions. BAM, border-associated macrophage. (H) Dot plot of interaction scores for ligand-receptor pairs between fibroblasts to reactive monocytes or inflammatory macrophages and operating signaling pathways in fibrinogen-depleted and control mice 6 days after PT. Dot size represents the log of the *p* value of aggregated scores. Dot color represents the ligand-receptor strength score from SCA analysis. ∗ ≥ 0.01 NATMI edge specificity. All data are shown as mean ± s.e.m. See also Figures S8–S12.

So far, we found that fibrinogen was immediately deposited in the perivascular space and triggers initial PVF activation after PT (Figures 1 and 2). Blood-derived monocytes (Ccr2+CD68^–^), inflammatory macrophages (Ccr2+CD68^+^) and activated perivascular macrophages (CD206+CD68^+^) in the perivascular space (Figures 3A, 3B, and S8) are present in the cortical ischemic stroke area within the first days after PT, coinciding with massive fibrinogen deposition (Figures 1B and 1C). Fibrinogen triggers inflammatory and pro-oxidant activation of microglia and peripheral immune cells via binding to CD11b/CD18.^19^ Next, we analyzed whether fibrinogen-induced myeloid cell activation contributes to initial PVF activation and fibrotic scar formation after PT. The contribution of neuroinflammation to fibrotic scar formation has been described in animal models of multiple sclerosis^11^ and spinal cord injury,^34^ but not stroke. Therefore, we depleted myeloid cells with the colony-stimulating factor 1 receptor (CSF1R) inhibitor PLX5622 (Figures S9A–S9C) and revealed a reduced periostin protein expression by 47% and a non significant reduced Col I expression by 30%, compared to vehicle-treated control mice at day 7 after PT (Figure 3C). Thus, myeloid cells revealed a contribution to fibrotic scar formation after cortical PT.

Next, we analyzed whether fibrinogen-induced myeloid cell activation, including perivacular macrophages, contributes to initial PVF activation. Perivascular macrophages and PVF share the same compartment in homeostatic conditions,^35^^,^^36^ and early activated perivascular macrophages (Figures 3B and S8) might be critical in shaping initial PVF responses after PT (Figures S10A and S10B). So far, there are no available pharmacological or genetic tools for a specific depletion of these cells that allow us to carefully evaluate their role in PVF activation. Therefore, we crossed the Fib^γ390–396A^ mouse line, which expresses mutant fibrinogen that retains normal clotting function but lacks the fibrinogen γ_390–396_ binding motif to CD11b/CD18,^37^ with the *collagen1α1-EGFP* reporter mouse line to generate *collagen1α1-EGFP:Fib*^*γ390–396A*^ mice to test early fibrinogen-induced myeloid cell activation, including early perivacular macrophage activation (Figures 3B and S8), on initial PVF activation. Immunolabeling for Col I and periostin in the *collagen1α1-EGFP:Fib*^*γ390–396A*^ fibroblast-reporter mouse line revealed that Col I expression in GFP+ PVFs was reduced by 45% and periostin expression was not significantly reduced by 30% at day 3 after PT (Figure 3D). This showed that early fibrinogen-induced myeloid cell activation, including the early activated perivascular macrophages, contributed to the initial PVF activation in the perivascular space after PT.

To characterize the contribution of fibrinogen to PVF-myeloid cell-cell interactions and fibrotic scar formation after PT, we performed scRNA-seq of the lesion core 6 days after PT and analyzed PVF-myeloid cell interaction in fibrinogen-depleted and control mice (Figure 3E). We obtained 3318 macrophages, 1515 monocytes, and 2634 fibroblasts in addition to brain parenchymal, vascular, inflammatory, and other mesenchymal cells (Figures 3F and S11A–S11D). To gain mechanistic insight into PVF-myeloid cell interaction in fibrinogen-depleted mice after distant cortical injury, we used the LIANA v0.1.12 package to evaluate interaction scores on the basis of the average expression levels of known ligands and receptors in two distinct cell populations.^38^ Interestingly, fibrinogen depletion drastically reduced the PVF-inflammatory macrophage interaction and increased PVF-monocyte interaction, suggesting that fibrinogen-triggered peripheral immune cell activation in the lesion core fosters cellular interactions with PVFs (Figures 3G, S12A, and S12B). The highest interaction scores between PVFs and inflammatory macrophages in the untreated condition included signaling pathways associated with the fibroblast receptors EGFR, SDC2, and integrin receptors, which have been described to regulate fibroblast proliferation and activation in other organs^39^^,^^40^^,^^41^ (Figure 3H). Interestingly, in ancrod-treated animals, these signaling pathways were depleted in inflammatory macrophages but gained in monocytes (Figure 3H), suggesting fibrinogen-driven alterations in PVF-myeloid cell interactions after cortical PT.

Overall, we revealed that immediate fibrinogen deposition in the perivascular space triggers initial PVF activation with a contribution of fibrinogen-induced myeloid cells and that fibrinogen alters PVF-myeloid cell interactions, potentially altering PVF and myeloid cell identity contributing to fibrotic scar formation.

### Fibrinogen depletion abrogates PVF-astrocyte interactions and increases neuronal plasticity at the lesion border after PT

**Figure 4:**
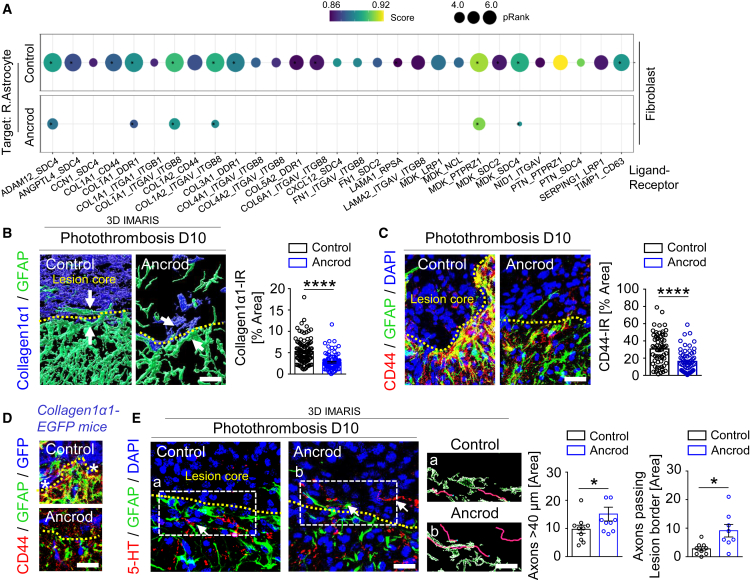
Fibrinogen depletion alters PVF-astrocyte interactions and improves neuronal plasticity at the lesion border after PT (A) Dot plot of interaction scores for ligand-receptor pairs between fibroblasts and reactive astrocytes and operating signaling pathways 6 days after PT in ancrod and control mice. (B) 3D reconstruction of collagen1α1 (blue) and GFAP (green) in the lesion border of fibrinogen-depleted (ancrod) and control mice 10 days after PT. White arrows indicate collagen1α1 and GFAP contact. Scale bar, 50 μm. Quantification of collagen1α1-immunoreactivity (IR) in the lesion border (*n* = 9 mice, 104 astrocytes, control; 98 astrocytes, ancrod). ∗∗∗∗*p* < 0.0001 by Mann-Whitney U test. (C) Representative images of CD44 (red) and GFAP (green) in the lesion border in fibrinogen-depleted and control mice 10 days after PT. Scale bar, 20 μm. Quantification of CD44–IR in the lesion border (*n* = 8 mice; 64 astrocytes, control; 62 astrocytes, ancrod). ∗∗∗∗*p* < 0.0001 by Mann-Whitney U test. (D) Representative images of CD44 (red), GFAP (green) and GFP (blue) in the lesion border in fibrinogen-depleted and control *collagen1α1-EGFP* mice 10 days after PT. Stars indicate PVF–CD44+ astrocyte colocalization. Scale bar, 20 μm. (E) Representative images of 5–HT (red) and GFAP (green) at the lesion border area of fibrinogen-depleted and control mice 10 days after PT, followed by 3D reconstruction of images (right). White arrows indicate serotonergic axons. Scale bars, 50 μm (overviews), 20 μm (magnifications). Quantification of serotonergic axons longer than 40 μm at the lesion border area (*n* = 10 mice). ∗*p* < 0.05 by Mann-Whitney U test. Quantification of serotonergic axons passing the lesion border (*n* = 10 mice, control; *n* = 9 mice, ancrod). ∗*p* < 0.05 by Student’s *t* test. All data are shown as mean ± s.e.m. See also Figure S13.

To investigate the contribution of fibrinogen-induced PVF activation on regeneration after PT, we focused on the interaction between PVFs and astrocytes, potentially altering astrocyte lesion border formation and neurorepair. Border-forming astrocytes are uniquely positioned precisely at the interface to the non-neural fibrotic lesion core^22^^,^^42^ (Figure S13A), and the fibrotic cell-astrocyte interface at the lesion border contributes to the chronic failure of axon regeneration.^43^ Interestingly, cell-cell interaction pathway analysis revealed a drastically reduced interaction between PVFs and astrocytes in fibrinogen-depleted mice (Figures 3G and S13B). In control mice, the highest PVF-astrocyte ligand-receptor interaction scores were associated with the ligands ADAM12, ANGPTL4, MDK, and TIMP1, and different collagen signaling pathways (Figure 4A), and in fibrinogen-depleted mice, these reactivity pathways were largely reduced or abolished with an overall reduction in cell-cell signaling (Figure 3G).

Fibroblast-secreted Col I mediates the interaction with astrocytes, inducing their activation.^44^ Thus, fibrinogen-induced PVF activation and Col I expression and secretion might regulate lesion border formation and impact neuronal regeneration. Fibrinogen depletion reduced Col I deposition contacting GFAP+ astrocytes at the lesion border by ∼50% at day 10 after PT, compared to the control group (Figure 4B). PVF-astrocyte ligand-receptor interaction scores showed that fibrinogen depletion drastically reduced PVF-astrocyte Col I-CD44 interaction (Figure 4A), suggesting that fibrinogen-triggered PVF–secreted Col I induces astrocyte activation and lesion border formation via CD44. Importantly, immunolabeling confirmed that fibrinogen depletion resulted in a reduced CD44 expression by GFAP+ astrocytes at the lesion border at day 10 after PT, compared to the control group (Figure 4C), and fibrinogen-depletion drastically reduced the colocalization of PVF Col I expression with astrocyte CD44 expression at the lesion border (Figure 4D). These data suggest that fibrinogen induces PVF activation and Col I expression and secretion and contributes to astrocyte activation and inhibitory lesion border formation.

Finally, we investigated whether fibrinogen-induced cortical fibrosis and astrocyte lesion border formation affects neurorepair. Remarkably, fibrinogen depletion by ancrod-treatment increased the NeuN+ cell number in the cortical ischemic stroke penumbra by ∼100%, compared to control mice (Figure S13C). Finally, immunolabeling for 5–hydroxytryptamine–positive (5–HT)+ nerve terminals at the lesion border area and labeling serotonergic fiber sprouting^45^ revealed an increase in axon length and axons passing the lesion border by ∼55% and ∼230%, respectively, in fibrinogen-depleted mice, compared to control (Figure 4E). Overall, these results reveal that cortical injury-induced fibrinogen deposition in the perivascular space induces PVF activation and fibrotic scar formation, limiting neurorepair after PT.

## Discussion

Understanding how cortical injury and the release of blood-derived factors regulate PVF activation, migration, and fibrotic scar formation is a fundamental and unresolved question. Our results suggest that, immediately upon cortical injury with vasculature permeability, blood-derived proteins (e.g., fibrinogen) alter the perivascular space ECM composition, orchestrating PVF cell signaling and cell-cell interaction, leading to fibrosis.

We suggest the following working model in CNS disease. (1) A cortical brain injury-induced vasculature rupture allows the deposition of vasculature-derived proteins, including fibrinogen, altering the ECM composition in the perivascular space. (2) Fibrinogen deposition in the perivascular space promotes β1 integrin-induced PVF activation and Col I expression and deposition. (3) Fibrinogen-induced PVF activation increases CD44 expression and PVF overt migration away from the vasculature. (4) Fibrinogen-induced myeloid cell activation contributes to PVF activation. (5) Fibrinogen deposition alters the PVF–astrocyte interaction, inducing astrocyte lesion border formation and hindering neuronal regeneration. Overall, we suggest that fibrinogen acts as an initial trigger of PVF activation within the perivascular space to orchestrate fibrotic scar formation after cortical brain injury.

Stroke can induce a chronic, non-healing pathology driven by stromal cells depositing an excess of ECM proteins and impeding CNS repair.^5^^,^^8^^,^^46^ PVFs in the perivascular space once activated in CNS disease, such as after stroke, proliferate, migrate away from the vasculature and express and secrete an excess of Col I, contributing significantly to fibrotic scar formation. We previously found that blood-derived fibrinogen leaks into the CNS immediately after injury and disease, correlating with areas of glial scarring and inflammation.^17^^,^^19^^,^^22^ Although fibrinogen is immediately deposited in the brain after vasculature rupture and although the perivascular space location of PVFs associated with larger blood vessels was known, a detailed anatomical description of fibrinogen provisional matrix deposition in relation to fibrotic cells after stroke was lacking. Here, we demonstrate that, after cortical stroke, fibrinogen is deposited in the perivascular space and colocalized with PVF and other perivascular cells, rapidly and drastically changing the ECM composition in the perivascular space. Interestingly, altered PVF activity and ECM accumulation in enlarged perivascular spaces has been observed in patients in the early stages of ALS.^47^ Future experiments should address whether early fibrinogen deposition in the perivascular space in different CNS diseases also affects cerebrovascular structures and induces fibrosis, contributing to CNS disease pathology.

The neuropathological effects of fibrinogen in the nervous system are numerous and include induction of astrocyte and myeloid cell activation. Fibrinogen triggers astrocyte activation by promoting the availability of active TGF-β after vascular damage.^17^ Fibrinogen-bound latent TGF-β interacts with local perivascular astrocytes. This interaction leads to active TGF-β formation, which induces reactive astrocytosis by regulating the TGF-β/Smad signaling pathway, resulting in deposition of inhibitory chondroitin sulfate proteoglycans (CSPGs).^17^ Here, we show that fibrinogen deposition in the perivascular space, via β1 integrin signal transduction, induces PVF activation and Col I expression and secretion. Thus, local provisional fibrin matrix functions as initial trigger of astrocyte^17^ and PVF activation (this study), initiating astrocyte activation, lesion border formation and fibrotic scar formation in the CNS. Fibrinogen contains multiple non-overlapping binding motifs for different receptors, including the tripeptide Arg-Gly-Asp (RGD) sequence on its alpha chain, which serves as a binding site for integrins.^16^^,^^48^ Future studies will further elucidate the site of fibrinogen-β1 integrin interaction and its role as initial trigger in fibrotic scar formation.

Inflammation contributes to fibrotic scar formation in the CNS.^11^ Given their positioning in the perivascular space, PVFs likely receive their activation cues via an altered perivascular space composition and/or via a changed cell-cell signaling with other proximal neurovascular cell types, such as perivascular macrophages, leading to both enhanced PVF ECM and signaling molecule production and their activation in response to CNS injury and disease. Fibrinogen binding to the CD11b/CD18 integrin on monocytes and perivascular macrophages mediates inflammatory responses,^20^^,^^21^ resulting in their expression and secretion of pro-inflammatory cytokines (e.g., IFNγ) and growth factors (e.g., TGF-β) that might contribute to PVF activation and fibrosis. IFNγ-induced signaling pathway activation was described in CNS fibrotic cells in a mouse model of multiple sclerosis.^11^ Our study suggests that immediate fibrinogen deposition in the perivascular space initiates directly PVF activation via β1 integrin signaling and that, in addition, fibrinogen-induced myeloid cell activation and particularly fibrinogen-induced perivascular macrophage activation is contributing to PVF activation and fibrosis.

The fibrotic scar replaces lost parenchymal cells and is coordinated with the generation of the lesion border formed by reactive astrocytes.^22^^,^^42^^,^^49^ Molecular cues coordinating fibroblast-astrocyte interaction to form the lesion border in CNS disease are poorly described.^42^^,^^50^^,^^51^ Interfering with the Col I-reactive astrocyte interaction prevented astrocytic scar formation, thereby leading to improved axonal regrowth and better functional outcomes in a mouse model of spinal cord injury.^44^ Our study revealed that fibrinogen depletion reduced Col I expression and PVF–astrocyte Col I-CD44 interaction, suggesting that fibrinogen-triggered PVF-secreted Col I might induce astrocyte activation and lesion border formation via CD44. Therefore, fibrinogen cell responses on myeloid cells, PVFs and astrocytes might represent a potential therapeutic nexus for improving repair in CNS disease.

In conclusion, we report that fibrinogen regulates early PVF activation in the perivascular space, inhibitory ECM deposition and PVF-astrocyte interactions, suggesting that fibrinogen deposition promotes an inhibitory environment that leads to fibrotic scar formation. Our findings represent a significant advancement in exploring the potential of fibrinogen reduction (anticoagulant therapy) or the modulation of fibrinogen-driven signaling pathways in PVFs for improving regenerative processes. We propose a therapeutic approach targeting the interaction between fibrinogen and PVFs to reduce fibrotic scar formation and minimize the deposition of inhibitory ECM. Future studies should describe the fibrinogen domain inducing PVF integrin signaling. Strategies to modulate fibrinogen-PVF interaction may be a promising therapeutic approach to reduce PVF activation and Col I expression and deposition promoting brain repair after stroke and, potentially, in other CNS diseases.

### Limitations of the study

Our findings suggest that fibrinogen triggers PVF activation in a mouse model of cortical ischemic stroke. A limitation of our study is that we used photothrombotic ischemia (PT) as mouse model of human stroke. However, photothrombotic damage slightly differs from human stroke, as described under “Limitations of the technique” in Labat-gest andTomasi, 2013.^52^ We used this model in our study, as this technique produces infarction of small size and clearly defined boundaries, which is highly advantageous for precise and reproducible cell characterization. Furthermore, early endothelial damage and vasculature-derived fibrinogen extravasation allowed us to study the fibrinogen effect on PVF activation. Finally, the major reason to choose PT as the stroke model in this study is that other stroke models produce larger lesions, which, combined with fibrinogen depletion, may cause severe hemorrhages leading to mouse death. Another limitation of our study is that we performed scRNA-seq to analyze PVF-scar cell interaction at day 6 after PT, but not at day 3 after PT, when PVF get first activated in the perivascular space. The low number of PVF in the lesion core 3 days after PT was a limiting factor for our sc-RNA-seq analysis. Third, while we show direct effects of fibrinogen on meningeal fibroblasts *in vitro*, studying its effects on PVF *in vitro* is challenging, as we were not able to culture FACS-isolated PVF of healthy mice. Fourth, treatment of primary fibroblasts with fibrinogen was not altering their migration, as this assay might not fully recapitulate the complex effects of fibrinogen on PVFs and their cell-cell communication *in vivo*. Lastly, while we show a contribution of fibrinogen-induced myeloid cells to PVF activation, we are currently not able to dissect a contribution of fibrinogen-induced perivascular macrophages to PVF activation, as there are currently no available pharmacological or genetic tools for the specific depletion of these cells that allow us to carefully evaluate their role.

## Resource availability

### Lead contact

Requests for further information and resources should be directed to and will be fulfilled by the lead contact, Christian Schachtrup (christian.schachtrup@anat.uni-freiburg.de).

### Materials availability

All mouse lines generated in this study are available from the lead contact with a completed materials transfer agreement.

### Data and code availability

• Data: The scRNA-seq data generated in this study were deposited in the NCBI Gene Expression Omnibus database under accession code GSE291902 and are publicly available as of the date of publication.
• Code: The code used to analyze the data in this study was deposited under Github and is publicly available at https://github.com/CoreyFehlberg/2024_Schatraup_Photothrombosis as of the date of publication.
• Additional information: Any additional information required to reanalyze the data reported in this paper is available from the lead contact upon request.

## Acknowledgments

We thank Meike Ast–Dumbach and Emina Deumic for technical assistance, J. Carrol for graphics, Gary Howard for editorial assistance, and the 10.13039/501100002714University of Freiburg Live Imaging Center (LIC) for microscopy support. We thank the University of Miami Hussman Institute of Human Genetics Sequencing Core for their assistance with single cell RNAseq. This study was supported by the Hannelore Kohl fellowship to J.C.M.S., the European Stroke Research Foundation (ESRF), the Else Kröner–Fresenius–Stiftung 2019_A53, and the DFG grant SCHA 1442/8-3 to C.S.

## Author contributions

J.C.M.S. performed the majority of the experiments. C.F. performed surgeries, tissue collection, analysis for the scRNA-seq experiments, and contributed to writing the manuscript. P.C. contributed to surgeries, immunohistochemical analysis of tissue sections, and imaging. J.N.N. and F.G.J. contributed to surgeries and performed the immunohistochemical analysis of tissue sections. P.M. contributed to cell culture and western blotting. F.B. contributed to the experimental design, data analysis, and interpretation. J.K.L contributed to surgeries, tissue collection, experimental design, data analysis and interpretation, and writing the manuscript. C.S. designed the study, analyzed the data, coordinated the experimental work and wrote the manuscript, with contributions from all authors. All co-authors have read and approved the final version of the manuscript.

## Declaration of interests

The authors declare no competing interests.

## STAR★Methods

### Key resources table

**Table:** 

REAGENT or RESOURCE	SOURCE	IDENTIFIER
**Antibodies**		

Rabbit polyclonal anti–5HT	Immunostar	Cat#20080; RRID:AB_572263
Rat monoclonal anti–Active integrin β1 (clone 9EG7)	BD Biosciences	Cat#550531; RRID:AB_393729
Rabbit polyclonal anti–AQP4	Santa Cruz	Cat#sc–20812; RRID:AB_2274338
Rabbit monoclonal anti–αSMA (clone EPR5368)	Abcam	Cat#ab124964; RRID:AB_11129103
Mouse monoclonal anti–CCR2 (clone 3G7)	Novus	Cat#NBP2–35334; RRID:AB_3294607
Goat polyclonal anti–CD31	R&D Systems	Cat#AF3628; RRID:AB_2161028
Rabbit polyclonal anti–CD44	Abcam	Cat#ab157107; RRID:AB_2847859
Rat anti–CD44 monoclonal antibody (clone IM7)	Invitrogen	Cat#14–0441–81; RRID:AB_467245
Rat monoclonal anti–CD68 (Clone FA-11)	Bio–Rad	Cat#MCA1957; RRID:AB_322219
Rabbit polyclonal anti–CD206	R&D Systems	Cat#AF2535; RRID:AB_2063012
Rabbit monoclonal anti–Collagen1α1 (Clone E8F4L)	Cell Signaling Technology	Cat#72026; RRID:AB_2904565
Sheep polyclonal anti–Fibrinogen	USBiological	Cat#F4203–02F; RRID:AB_2103506
rabbit recombinant monoclonal anti–GAPDH (Clone 14C10)	Cell Signaling Technology	Cat#2118; RRID:AB_561053
Rat monoclonal anti–GFAP (Clone 2.2B10)	Invitrogen	Cat#13–0300; RRID:AB_86543
Chicken polyclonal anti–GFP	Abcam	Cat#ab13970; RRID:AB_300798
Rabbit polyclonal anti–Iba1	Wako	Cat#019–19741; RRID:AB_839504
Rat IgG2b kappa isotype control (Clone eB149/10H5)	Invitrogen	Cat#14–4031–81; RRID:AB_470098
GSL I anti–Isolectin–b4	Vector Laboratories	Cat#RL1102; RRID:AB_2336492
Rabbit recombinant monoclonal anti–Ki67 (clone SP6)	Abcam	Cat#ab16667; RRID:AB_302459
Rabbit monoclonal anti–NeuN (clone EPR12763)	Abcam	Cat#ab177487; RRID:AB_2532109
Rabbit polyclonal anti–Pan–laminin	Sigma	Cat#L9393; RRID:AB_477163
Goat polyclonal anti–Periostin	R&D Systems	Cat#AF2955; RRID:AB_664123
Rabbit polyclonal anti–pSMAD2 (Ser465/467)	Cell Signaling Technology	Cat#3101; RRID:AB_331673
Rabbit recombinant monoclonal anti–TGF–β1 (clone EPR21143)	Abcam	Cat#ab215715; RRID:AB_2893156
Mouse monoclonal anti–Vinculin (Clone 7F9)	Invitrogen	Cat#14–9777–82; RRID:AB_2573028
Alexa Fluor 488-conjugated AffiniPure donkey Anti-Chicken IgY	Jackson ImmunoResearch	Cat#703-545-155; RRID:AB_2340375
Alexa Fluor 488-conjugated AffiniPure donkey Anti-Goat IgG	Jackson ImmunoResearch	Cat#705-545-147; RRID:AB_2336933
Alexa Fluor 488-conjugated AffiniPure donkey Anti-Rat IgG	Jackson ImmunoResearch	Cat#712-545-150; RRID:AB_2340683
Alexa Fluor 488-conjugated AffiniPure donkey Anti-Rabbit IgG	Jackson ImmunoResearch	Cat#711-545-152; RRID:AB_2313584
Alexa Fluor 594-conjugated AffiniPure donkey Anti-Goat IgG	Jackson ImmunoResearch	Cat#705-585-147; RRID:AB_2340433
Alexa Fluor 594-conjugated AffiniPure donkey Anti-Rabbit IgG	Jackson ImmunoResearch	Cat#711-585-152; RRID:AB_2340621
Alexa Fluor 594-conjugated AffiniPure donkey Anti-Sheep IgG	Jackson ImmunoResearch	Cat#713-585-147; RRID:AB_2340748
Alexa Fluor 594-conjugated AffiniPure donkey Anti-Mouse IgG	Jackson ImmunoResearch	Cat#715-585-150; RRID:AB_2340854
Alexa Fluor 594-conjugated AffiniPure donkey Anti-Rat IgG	Jackson ImmunoResearch	Cat#712-585-153; RRID:AB_2340689
Alexa Fluor 647-conjugated AffiniPure donkey Anti-Rat IgG	Jackson ImmunoResearch	Cat#712-605-150; RRID:AB_2340693
Alexa Fluor 647-conjugated AffiniPure donkey Anti-Rabbit IgG	Jackson ImmunoResearch	Cat#711-605-152; RRID:AB_2492288
HRP-linked goat anti–rabbit IgG	Cell Signaling Technology	Cat#7074; RRID:AB_2099233

**Chemicals, peptides, and recombinant proteins**		

Tamoxifen	Sigma Aldrich	Cat#T6548
Ancrod	NIBSC	Cat#74/581
AIN–76A diet and control diet	Plexxikon	N/A
Rose Bengal	Sigma–Aldrich	Cat#330000
Collagenase II	Worthington Biochemical	Cat#LS0004174
PP2	Sigma	Cat#P0042
PDGF–BB	Peprotech	Cat#315–18
Hirudin	Sigma	Cat#H0393
Fibrinogen	Sigma	Cat#341578

**Critical commercial assays**		

Neural Tissue Dissociation Kit–P	Miltenyi Biotec	Cat#130–092–628
Dual Index Kit TT (Set A)	10X Genomics	Cat#PN-1000215
Cells–to–CT lysis solution	ThermoFisher	Cat#4391851C
ApopTag Red *In Situ* Apoptosis Detection Kit	Merck	Cat#S7165

**Deposited data**		

scRNA–Seq dataset	This paper	NCBI Gene Expression Omnibus database: GSE291902
Code for scRNA–Seq data analysis	This paper	Github: https://github.com/CoreyFehlberg/2024_Schatraup_Photothrombosis

**Experimental models: Cell lines**		

NIH3T3cells	ATCC	Cat#CRL-1658; RRID:CVCL_0594

**Experimental models: Organisms/strains**		

C57BL/6N mice	Charles River	N/A
*Collagen1α1–EGFP* mice	Donated by PD Dr. Ingmar Mederacke	N/A
*Collagen1α2–Cre*^*ERT2*^ mice	The Jackson Laboratory	Cat#029567; RRID:IMSR_JAX:029567
*Rosa26–EYFP* mice	The Jackson Laboratory	Cat#006148; RRID:IMSR_JAX:006148
*Fib*^*γ390–396A*^ mice	Donated by Prof. Katerina Akassoglou	N/A

**Oligonucleotides**		

Collagen1α1 forward primer: 5′ CCTCAGGGTATTGCTGGACAAC 3′	Eurofins	N/A
Collagen1α1 reverse primer: 5′ ACCACTTGATCCAGAAGGACCTT 3′	Eurofins	N/A
Periostin forward primer: 5′ CAGCAAACCACTTTCACCGACC 3′	Eurofins	N/A
Periostin reverse primer: 5′ AGAAGGCGTTGGTCCATGCTCA 3′	Eurofins	N/A
GAPDH forward primer: 5′ CAAGGCCGAGAATGGGA 3′	Eurofins	N/A
GAPDH reverse primer: 5′ GGCCTCACCCCATTTGAT 3′	Eurofins	N/A

**Software and algorithms**		

ImageJ	NIH	RRID:SCR_003070
GraphPad Prism v6	GraphPad	RRID:SCR_002798
Leica Application Suite X	Leica	RRID:SCR_013673
Zen v3.0 Blue edition	Zeiss	RRID:SCR_013672
BD FACSDiva v9	BD Biosciences	RRID:SCR_001456
Summit v6.3	Beckman Coulter Life Sciences	RRID:SCR_018893
IMARIS v9	BitPlane	RRID:SCR_007370
Cell Ranger’s v7.1.0	10x Genomics	RRID:SCR_021160
STAR	N/A	RRID:SCR_004463
SingleR	N/A	RRID:SCR_023120
LIANA v0.1.12 package	N/A	N/A

### Experimental model and study participant details

#### Cell culture

NIH3T3cells (male, ATCC, Cat#CRL-1658) and primary murine meningeal fibroblasts (female and male) were cultured in DMEM supplemented with 10% FBS and 1% penicillin-streptomycin, and incubated at 37°C and 5% CO2. All cells were passaged at 90% confluency. Media was replenished every 2 days.

#### Animals

C57BL/6N mice (Charles River) and transgenic mice (C57BL/6N background) were used. For the analysis of PVFs, *Collagen1α1–EGFP*^12^ reporter mice, donated by PD Dr. Ingmar Mederacke, and *Collagen1α2–Cre*^*ERT2*^^25^ (The Jackson Laboratory, catalog #029567) were used, and *Collagen1α2–Cre*^*ERT2*^ mice were crossed with *Rosa26–EYFP*^53^ (The Jackson Laboratory, catalog #006148) mice, resulting in *Collagen1α2–Cre*^*ERT2*^*:Rosa26–EYFP* mice. For the analysis of fibrinogen–activated myeloid cells, *Fib*^*γ390–396A*^ mice,^37^ donated by Prof. Katerina Akassoglou, were crossed with *Collagen1α1–EGFP* mice to generate *Fib*^*γ390–396A*^*:Collagen1α1–EGFP* mice. 8–12–weeks–old mice of both genders were used. Animals were housed under Institutional Animal Care and Use Committee guidelines in a temperature and humidity–controlled facility with a 12 h light–12 h dark cycle and *ad libitum* feeding. All animal experiments were approved by the Federal Ministry for Nature, Environment, and Consumer Protection of the state of Baden–Württemberg (G17/89, G21/056) and were performed in accordance with the respective national, federal, and institutional regulations.

### Method details

#### Tamoxifen treatment

For the induction of Cre recombinase in the *Collagen1α2–Cre*^*ERT2*^*:Rosa26–EYFP* mouse line*,* 6–weeks–old mice were intraperitoneally injected with a daily dose of tamoxifen (TAM, Sigma Aldrich, T6548) (80 mg/kg body weight, dissolved in corn oil (Sigma Aldrich)) for 2 consecutive days.^54^ Photothrombotic ischemia (PT) was performed 14 days after the last injection.

#### Ancrod treatment

To investigate the fibrinogen–induced effects on PVF activation and fibrotic scar formation, mice were depleted of fibrinogen with ancrod (NIBSC, 74/581). Briefly, mice received 2.4 U ancrod, 2.0 mg per kg body weight, or saline solution (control), per day via osmotic minipumps (Alzet, Model: 2002, 0.5 μL/h) implanted subcutaneously in their back 2 days before stroke induction. Ancrod was secreted for the duration of the experiment until the mice were perfused.

#### Myeloid cells depletion

For pharmacological ablation of brain myeloid cells, mice were fed a PLX5622–formulated AIN–76A diet (1.2 g PLX5622 per kilogram of diet, Plexxikon) *ad libitum,*^55^ with normal AIN–76A diet (Plexxikon) as control. Mice were fed with the PLX5622 diet for 12 days. PT was performed 5 days after the start of the PLX5622 diet, and mice were sacrificed 7 days after PT.

#### Laser–induced CNV model

The laser–induced CNV model was conduced as previously described.^27^ Briefly, *Collagen1α1–EGFP* mice were anesthetized with an intraperitoneal injection of 10% ketamine and 2% xylazine. Pupils were dilated using 0.5% tropicamide eye drops (Mydriaticum Stulln, Pharma Sutlln GmbH), and the eyes were kept moist with Dexpanthenol Eye Gel (Corneregel 50 mg/g, Bausch + Lomb) during the procedure. Once the pupils were dilated, mice were placed in front of an argon laser (VISULAS 532s, ZEISS). A coverslip with Dexpanthenol Gel was positioned on the eye to act as a contact lens, transforming the curved cornea into a flat surface. Three laser burns were placed at equal distances from the optic disc induced by the laser with a wavelength of 532 nm (power: 150 mW, duration: 100 ms, spot size: 100 μm). The presence of a bubble at the site of the laser burn confirmed retinal pigment epithelial rupture, a critical step for inducing CNV. Three laser spots per eye were applied. At day 7 post–laser treatment, CNV lesions were analyzed. Eyes were enucleated, fixed in 4% paraformaldehyde, and dissected to prepare choroidal flatmounts.

#### Stroke model

PT was used to induce stroke in the cortex of adult mice.^24^^,^^52^ Briefly, 20 min after injection of the photosensitive dye Rose Bengal (Sigma–Aldrich, 330000; 10 μL/g body weight, intraperitoneal), a cold light illuminator was positioned stereotaxically to deliver light (150 W) to the cortex (AP, 0 mm; ML, −2.4 mm, according to Paxinos and Watson). The region of interest (4–mm diameter) was illuminated for 6 min, and after the light exposure ended, the wound was sutured.

#### Cortical lesion microdissection

Injury sites were isolated 6 days after injury. Mice were anesthetized with Avertin and transcardially perfused with ice–cold, oxygenated artificial cerebrospinal fluid. The meninges were removed, and the injury site and surrounding spared tissue were excised in an approximate 5 mm by 5 mm square, to a depth of up to but not including the corpus callosum. All steps were performed with pre–chilled solutions on ice or at 4°C unless otherwise noted. The tissue was roughly chopped with a razor blade, resuspended in Cutting Solution, then centrifuged at 300g for 2 min at room temperature (RT). After aspirating the supernatant, the sample was processed using the Miltenyi Neural Tissue Dissociation Kit–P (cat #130–092–628), according to manufacturer’s guidelines. During the second enzyme digestion step, cells were triturated 10 times each in 1,000, 750, and 500 μM fire–polished Pasteur pipettes. After the final centrifugation, supernatant was aspirated, and cells were resuspended in 1 mL Red Blood Cell Lysis buffer and incubated at RT for 1 min. This was stopped with 5 mL MACS Buffer and centrifuged at 300g for 5 min. The resulting single–cell pellet was then incubated with Miltenyi Myelin Removal Beads II, according to manufacturer’s guidelines. Cell–bead suspensions were passed through an equilibrated LS column that was then washed with 2 mL of MACS buffer. After centrifugation at 300*g* for 5 min and supernatant aspiration, cells were resuspended in 50 μL of FACS buffer and kept on ice until sequencing.

#### Single–cell RNA sequencing

Single–cell suspensions were checked for viability, density, and purity using AOPI Stain imaged on an Nexcelom K2 Cellometer. Samples were diluted to ∼1,000 cells/μL and had a minimum viability of 80%. ∼6,000 cells per sample were sequenced with ∼100,000 mean reads and ∼13,000 median UMI counts per cell. Sample libraries were prepared with the 10X Chromium Next GEM Single Cell 3′ Reagent Kits v3.1 (Dual Index) and multiplexed on the sequencer with Dual Index Kit TT (Set A, 10X Genomics, Cat#PN-1000215), according to manufacturer’s protocols. These libraries were then sequenced using paired–end sequencing on eight channels of an Illumina Novaseq X 10–billion–read flow cell with a 300–cycle kit. The resulting sequences were then aligned and filtered using Cell Ranger’s v7.1.0 count pipeline to generate gene–barcode count matrices. In brief, base call files for each sample were demultiplexed into FASTQ reads and then aligned to the mouse mm39 reference genome using the STAR splice–aware aligner. Confidently mapped exonic and intronic reads were further aligned to annotated transcripts. After barcode and UMI quality filtering and sequence correction, reads were grouped by barcode, UMI, and gene annotation and used for UMI counting. The output was a count matrix containing all UMI counts for every droplet. Sequence alignment and transcript counts were performed using Cell Ranger version 7.1.0 with the 10X Genomics pre–built Cell Ranger mouse reference package, from GRCm39.

#### Single–cell RNA sequencing data quality control and annotation

The raw feature matrices were processed individually as follows. Empty droplets were filtered using both barcode UMI ranking (removing droplets that fall below the inflection point) and the emptyDrops function from the DropletUtils package (setting the lower bounds to the lower knee of the UMI ranking and retaining points above the inflection point). Next, the passing cells were filtered to be less than 30% mitochondrial genes. The number of counts and features were log10 transformed and filtered to remove cells greater than 3 median absolute deviations from the median of either metric. Scrublet was then used to remove doublets using the estimated doublet rates provided by 10X. Predicted doublets were validated by clustering the cells using Seurat’s default pipeline (with npcs = 20, dims = 1:20, resolution = 6) and checking for overlapping expression of known marker genes. Confirmed doublet clusters were removed, and any doublet clusters that segregated out after later integration of the datasets were also removed. Potential doublet clusters were not removed if the overlapping marker genes were from cells of the same niche (i.e., macrophages and neutrophils or endothelial cells and pericytes). The resulting datasets were then re– merged, normalized, and clustered, then annotated for broad cell types using expression levels of known marker genes, filtering with scGate using marker genes, and mapping to existing datasets^56^ with SingleR. The large dataset was then split into neuronal, immune and vascular niches and annotated for cellular subtypes in the same manner. Explicit integration with regression on metadata or other weighted integration methods were not performed as sample and treatment groups were visually well–integrated after dimensional reduction of the combined datasets. After annotation, cells associated with the injury site (reactive fibroblasts and macrophages) had proportions which trended higher in the fibrinogen–depleted animals and lower in the control animals, whereas cells associated with spared tissue (homeostatic astrocytes) were relatively equivalent. This is likely due to reduced fibrosis after ancrod treatment, leading to easier dissociation of cells at the injury site.

#### Ligand–receptor analysis

To assess cell–cell communication using single–cell data, we used the LIANA v0.1.12 package. Samples were split by treatment and grouped by cell subtypes. Groups were processed using natmi, connectome, logfc, and cellphonedb rankings and the Consensus resource (nperms = 50). Top connections were sorted by the lower 5th percentile of aggregate rank, upper 95th percentile of LRscore of the sca ranking, and upper 95th percentile of edge specificity of the natmi ranking.

#### Flow cytometry of PVFs

*Collagen1α1–EGFP* reporter mice were anesthetized with ketamine and xylazine, and transcardially perfused with PBS. Brains were immediately harvested and processed.^22^ Briefly, brains were placed in the dissection solution (0.8% glucose and 15 mM HEPES in HBSS) on ice. Meninges were removed using small forceps and a light microscope (Leica MZ10). The lesion core was further microdissected, chopped into small pieces, followed by enzymatic digestion with papain (Worthington, 50 units per mouse brain tissue) in PIPES solution [(120 mM NaCl, 5 mM KCl, 50 mM PIPES[Sigma–Aldrich]), 0.6% glucose, 1% pen/strep in water, pH adjusted to 7.6] for 10 min at 37°C, and mechanically dissociated with fire–polished glass Pasteur pipettes after adding ovomucoid (Worthington, 0.14 mg/mouse) and DNase I (Invitrogen, 100 units/mouse). Myelin debris was removed by centrifuging in 22% Percoll (Sigma–Aldrich) for 10 min at 4°C without brakes. Single cells were collected by centrifugation (176 g, 5 min) and re–suspended in DPBS containing 2% FBS. For theanalysis of the gene expression of *collagen1α1* and *periostin*, PVF were sorted based on GFP fluorescence using BD FACSAria Fusion (BD Biosciences) and MoFlo Astrios (Beckman Coulter Life Sciences) sorter at the Flow Cytometry Core at the Lighthouse Core facility (Freiburg, Germany) into Cells–to–CT lysis solution (ThermoFisher, Cat#4391851C). Cells were analyzed using the BD FACSDiva 9 (BD Biosciences) and Summit V6.3 (Beckman Coulter Life Sciences) software.

#### RNA isolation and quantitative PCR

RNA was isolated from primary PVFs and quantitative real–time PCR was performed.^57^

#### Infarct volume analysis

To measure the infarct volume, 40-μm thick coronal sections were cut from mouse brains 7 days after PT and then immunolabeled with an anti-GFAP antibody. The ischemic lesion was outlined with ImageJ on coronal sections spaced 200-μm apart, as previously described.^54^ Infarct volumes were derived by calculating the average infarct area between slices and multiplying by the distance between the start and end slices.

#### Immunohistochemistry

Mice were transcardially perfused with ice–cold saline, followed by 4% PFA in phosphate buffer under ketamine and xylazine anesthesia. Brain samples were removed, stored in 30% sucrose in phosphate buffer for 2 days, embedded in tissue freezing medium (Tissue–Tek), and frozen in a −80°C freezer. Brain samples were cut by cryosectioning (CM3050S Cryostat, Leica) in 14– or 40–μm coronal sections, mounted on superfrost slides, and immunohistochemistry was performed.^22^ Briefly, sections were permeabilized with PBS–triton 0.1% (14–μm–thick sections) or PBS–triton 0.3% (40–μm–thick sections) for 30 min, blocked in 5% BSA for 1 h and finally incubated overnight (14–μm– thick sections) or for 2 days (40–μm–thick sections) with primary antibody in PBS with 1% BSA. The following primary antibodies were used: rabbit anti–5HT (1:2000, Immunostar, 20080), rat anti–active integrin β1 (1:50, BD Biosciences, 550531), rabbit anti–AQP4 (1:50, Santa Cruz, sc–20812), rabbit anti–αSMA (1:500, abcam, ab124964), mouse anti–CCR2 (1:1000, Novus, NBP2–35334), goat anti–CD31 (1:500, R&D Systems, AF3628), rabbit anti–CD44 (1:500, abcam, ab157107), rat anti–CD68 (1:500, Bio–Rad, MCA1957), rabbit anti–CD206 (1:500, R&D Systems, AF2535), rabbit anti–collagen1α1 (1:200, Cell Signaling Technology, 72026S), sheep anti–fibrinogen (1:500, USBiological, F4203–02F), rat anti–GFAP (1:2000, Invitrogen, 13–0300), chicken anti–GFP (1:2000, abcam, ab13970), rabbit anti–Iba1 (1:500, Wako, 019–19741), anti–isolectin–b4 (1:500, Vector Laboratories, RL1102), rabbit anti–Ki67 (1:200, Abcam, ab16667), rabbit anti–NeuN (1:500, Abcam, ab177487), rabbit anti–pan–laminin (1:2000, Sigma, L9393), goat anti–periostin (1:300, R&D Systems, AF2955), rabbit anti–pSMAD2 (1:100, Cell Signaling Technology, 3101), rabbit anti–TGF–β1 (1:200, Abcam, ab215715) and mouse anti–vinculin (1:100, Invitrogen, 14–9777–82). Secondary antibodies used included donkey antibodies to rabbit, rat, guinea pig, mouse, sheep, and goat, conjugated with Alexa Fluor 488, 594 or 647 (1:200, Jackson ImmunoResearch Laboratories). Sections were cover–slipped with DAPI (Southern Biotechnology).

#### ApopTag assays

ApopTag assays were carried out using the ApopTag Red *In Situ* Apoptosis Detection Kit (Merck, Cat#S7165), according to the manufacturer’s instructions. Briefly, specimens were permeabilized in PBS containing 0.1% Triton–X 100 for 15 min, washed once with PBS for 5 min and subsequently incubated with ethanol:acetic acid (2:1) at −20°C for 5 min, followed by blocking and then primary and secondary antibody treatments, as above. Thereafter, specimens were treated with equilibration buffer for 10 min at RT, followed by treatment with a working strength TdT enzyme mixture for 1 h at 37°C in a humid chamber. The enzymatic reaction was stopped with the working–strength stop/wash buffer supplied with the kit. Specimens were then treated with anti–digoxigenin conjugate for 30 min at 37°C in a humid chamber, followed by washing steps and mounting with DAPI–Fluoromount.

#### Fibroblast isolation and culture

Meninges of cerebral cortices from neonatal C57Bl/6J mice (3–5 days of age) were dissected, enzymatically digested in 0.2% trypsin and 0.25% collagenase II (Worthington Biochemical, Cat#LS0004174) for 15 min at 37°C, and the tissue was triturated into single cells.^58^ The cell suspension was cultured with DMEM supplemented with 10% FBS and 1% pen/strep at 37°C in uncoated 60 cm^2^ tissue–culture dishes. After 7 days, meningeal fibroblasts were treated with 0.2% trypsin–EDTA for 5 min and re–seeded or plated for the outlined experiments.

#### Immunoblots

For Collagen1α1 and CD44 expression analysis, meningeal fibroblasts or NIH3T3cells were seeded at a density of 1 × 10^5^ cells/well in uncoated 12–well plates (Falcon) in DMEM supplemented with 10% FBS and 1% pen/strep at 37°C for cell attachment, then starved overnight, pretreated either with 10 μM PP2 (Sigma, Cat#P0042) or the same volume of DMSO (control, Sigma) for 1 h and treated either with 1 μm/mL hirudin (Sigma, Cat#H0393) for 15 min followed by 2.5 mg/mL fibrinogen (Sigma, Cat#341578), or left untreated (control). 30 ng/mL recombinant murine PDGF–BB (Peprotech, 315–18) was used as positive control. Protein extracts from meningeal fibroblasts were separated by electrophoresis on a 4–12% gradient. The following primary antibodies were used: rabbit anti–Collagen1α1 (1:1000, Cell Signaling Technology, 72026S), rabbit anti–CD44 (1:1000, abcam, ab157107) and rabbit anti–GAPDH (1:1000, Cell Signaling Technology, #2118). Blots were washed three times with TBS–T, incubated with peroxidase–labeled secondary antibodies (goat anti–rabbit IgG, Cell Signaling Technology, 1:5000), diluted in 5% nonfat milk in TBS–T for 1 h at room temperature, and washed again, followed by detection with chemiluminescence (ECL, GE Healthcare).

#### Fibroblast migration assay

Meningeal fibroblasts were seeded at a density of 2 × 10^4^ cells/well in uncoated 8–well culture slides (Falcon) with DMEM supplemented with 10% FBS and 1% pen/strep at 37°C until confluency. Hereafter, cells were pretreated overnight with anti–CD44 blocking monoclonal antibody IM7 (50 μg/mL, Invitrogen, 14–0441–81) or rat IgG2b kappa isotype control (50 μg/mL, Invitrogen, 14–4031–81) in DMEM in absence of FBS. Cells were treated with 1 μL/mL hirudin for 15 min, followed by 2.5 mg/mL fibrinogen for 1 day. Recombinant murine PDGF–BB (30 ng/mL, Peprotech, 315–18) served as positive control. A scratch was made in the monolayer with a pipette tip (200 μL) and migration of fibroblasts was evaluated 24 h later.

#### Immunocytochemistry

Meningeal fibroblasts were seeded (4 × 10^4^ cells/well) in fibrin–coated (250 μg/mL) 8–well culture slides (Falcon) in DMEM supplemented with 10% FBS and 1% pen/strep at 37°C and fixed 2 days later. Meningeal fibroblasts were rinsed with ice–cold PBS, fixed in 4% PFA for 15 min at 4°C, washed three times with PBS, permeabilized for 10 min at 4°C in PBS plus 0.2% Triton X–100 (by volume), blocked in PBS with 5% BSA for 30 min at 4°C, and washed three times in PBS. The primary antibodies used were rabbit anti–αSMA (1:500, abcam, ab124965) and chicken anti–GFP (1:2000, ab13970), and the secondary antibodies used included donkey antibodies to rat, rabbit and mouse conjugated with Alexa Fluor 488, 594 or FITC (1:200, Jackson ImmunoResearch Laboratories). The slides were mounted with DAPI Fluoromount–G.

#### Microscopy and imaging analysis

Images were acquired with a Leica TCS SP8 laser confocal microscope from 3 to 4 brain sections (per mouse) belonging to the plane of injury. Z–stacks were collected with a 0.5–1.0–μm step size and combined into maximum intensity projections with the LAS AF image–analysis software (Leica). For the overview images of the lesion core, tile–scan images were acquired with a 20X objective. For protein expression analysis, images containing PVFs in the lesion core were acquired with 63X oil objectives. For immunoreactivity (IR) analysis, images were saved in TIFF format. With ImageJ (NIH), images were converted into 16–bit images and thresholded. IR was calculated by the number of colored pixels (positively stained areas) divided by the total number of pixels (sum of positively and negatively stained area) in the imaged field. To analyze fibrinogen– and GFP–IR from overview images, regions of interest (ROIs) of 200 × 200 μm were drawn at an area of the lesion core adjacent to the lesion border and IR was measured. To analyze Col I–, periostin, CD44 and vinculin–IR in PVF after PT, ROIs were drawn based on GFP signal to delineate fibroblasts, and then IR was measured in the previously drawn ROIs. To analyze the GFP–IR in overview images 6 days after PT in ancrod or control mice, IR was measured in the center (1.5 mm diameter) of the lesion core. For the analysis of periostin- and Col I-IR in the lesion core of PLX5622–treated mice, IR was measured in tilescan images and values were normalized by the average of the control group. For the analysis of Iba1-IR in the lesion core and lesion rim of PLX5622–treated mice, the lesion core and the lesion rim (first 100 μm outside the lesion core) areas were manually delineated and IR was measured separately in each area from tilescan images. CCR2+ monocytes and CD206+ macrophages were counted manually in tilescan images of the lesion core. To analyze Col I in contact with GFAP+ cells and CD44 expression by GFAP+ cells 10 days after PT, 63x images were taken at 2 different sides of the lesion border. A random GFAP+ astrocyte directly positioned at the lesion border was manually delineated and IR was measured in that selected area. For the analysis of the number of GFP+ cells in the lesion core, cells were manually counted from tile–scan images of the whole lesion core, and the number of GFP+ cells was normalized by the lesion core area. For the analysis of the number of Ki67+ or ApopTag+ GFP+ cells in the lesion core, cells were manually counted from images taken from two different sides of the lesion core. For the analysis of laser–induced CNV mice, positive areas were measured as lesion sizes in pixels from images taken with a 20x objective. For the analysis of the expression of αSMA, αSMA–IR was measured in an ROI comprising 200 × 800 μm of the lesion core adjacent to the lesion border. For the analysis of the expression of αSMA by meningeal fibroblasts, αSMA–IR was measured in five random images taken by an Axio Imager M2 fluorescence microscope (Zeiss) of 8–well culture slides and normalized by the number of fibroblasts present in each image and relativized by the control group. Images were collected using the Zen Blue edition software (Zeiss). For the *in vitro* fibroblast migration assay, the wound area was photographed at *t* = 0 and *t* = 24 h with a light–field microscope (Primo Vert, Zeiss) coupled to a digital camera (Canon PC1252 DC 4.3V). The pictures were analyzed using ImageJ. The migration rate was determined by the wound area at the two times and relativized to the untreated group. For the analysis of CD206+ TGFβ1+ macrophages, cells were manually counted in three different sides of the lesion core. For neuronal survival analysis, images were taken with a 20x objective, and NeuN+cells were counted manually in a ROI comprising 150 × 900 μm in the lesion border.

#### Three–dimensional reconstructions

β1 integrin, vinculin and αSMA expression in PVFs, as well as, TGF–β1+CD206+ macrophages were represented as 3D reconstructed images. For fibroblast reconstruction and fibrinogen deposition in the perivascular space, images were acquired from 40–μm–thickness samples with a 63X objective. A total of four images containing PVFs were taken for each section. For fibrinogen deposition in the perivascular space, briefly, one surface for laminin and another for fibrinogen were created using the automatic surface rendering tool of Imaris. Applying a filter in the option mode, only fibrinogen colocalizing with laminin was measured, automatically excluding fibrinogen deposited in the parenchyma and in the vessel lumen. Based on this filter, the volume of fibrinogen was analyzed. For fibroblast reconstruction, using the Imaris software (Bitplane) the manual surface mode was selected with the Z–plane mode one. An imaginary surface was created based on the GFP+ signal to mask each PVF. Automatic surface mode was used on the masked channel to reconstruct the 3D structure of the PVF. Surface volume of the GFP soma was analyzed. For PVF overt migration analyses, images were taken from representative areas of the lesion core with a 10x dry objective, and 3D reconstruction of the surfaces was done for nuclei, GFP+ PVFs and CD31^+^ vessels. The statistical tool to measure the shortest distance to the closest vessel was used to compile the information about each PVF in the image. The rate of overt migration was established as the number of PVFs whose shortest distance to the closest vessel was higher than 5 μm divided by total number of PVFs. The length of migration was defined as the shortest distance to the closest vessel for every overtly migrated PVF. For perivascular macrophage activation analysis, one surface for CD206 and another for CD68 were created, and only CD68 colocalizing with CD206 was measured for each cell. Based on this filter, the volume of CD68 in CD206+ cells was analyzed. For 5-HT axon reconstruction using the IMARIS Filament Tracker, axons within 100 μm on both sides of the lesion border were manually traced using the AutoPath function. Subsequently, only axons longer than 40 μm were considered for the quantification. Importantly, for the quantification of axons passing the lesion border, all axons were considered regardless of their length. For pSMAD2 nuclear location analysis, 3D reconstruction of the surfaces was done for nuclei, GFP+ PVF and pSMAD2. The overlapping volume ratio between pSMAD2 and DAPI was calculated for each individual PVF.

### Quantification and statistical analysis

Data are shown as the means ± SEM. Differences between groups were examined with one–way ANOVAs for multiple comparisons, followed by Bonferroni corrections for comparison of means; differences between two experimental groups were assessed by non–parametric two–tailed unpaired Mann–Whitney tests or two–tailed unpaired Student's t test when the data followed a Gaussian distribution. Analyses were conducted in GraphPad Prism (GraphPad Software, Version 6.0, La Jolla, USA). Differences were considered significant when the *p*–value was <0.05. Statistical details of experiments can be found in the figure legends.
