# Supplementary material for: Fibrinogen triggers perivascular fibroblast activation in a mouse model of cortical ischemic stroke
Source: iScience. 2025 Oct 24;28(11):113834. doi: 10.1016/j.isci.2025.113834 (PMC12629924; doi:10.1016/j.isci.2025.113834)
Supplement: Document S1. Figures S1–S13 [file mmc1.pdf]

iScience, Volume *28*

Supplemental information

Fibrinogen triggers perivascular fibroblast activation in a mouse model of cortical ischemic stroke

Jose C. Martínez Santamaría, Corey Fehlberg, Pasquale Conforti, Jan N. Ness, Francesca Garafulic Justiniano, Pedro Manzitti, Felicitas Bucher, Jae K. Lee, and Christian Schachtrup

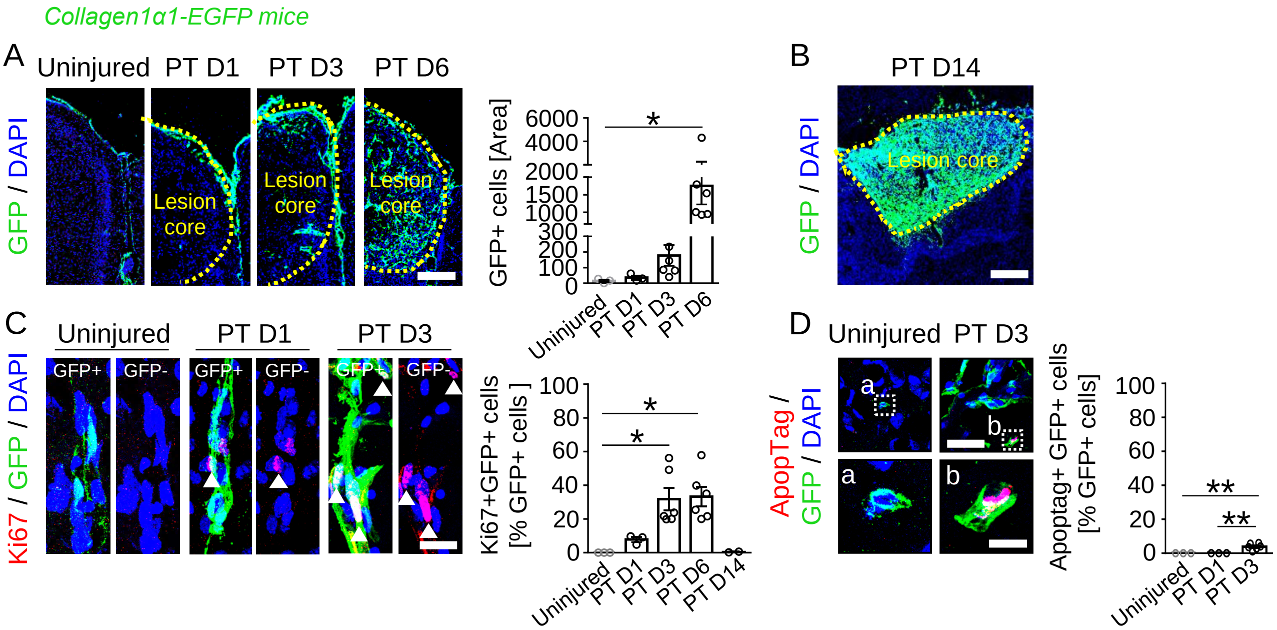

Figure S1 | PVF cell number increase in the lesion core after PT, related to Figure 1.

A. Representative images of GFP (green) in the lesion core 1, 3 and 6 days after PT, compared to uninjured mice. Scale bar, 150 µm. Quantification of GFP+ cells per area in the lesion core (n = 3 mice, uninjured and PT D1; n = 6 mice, PT D3 and PT D6). *P<0.05 by one-way ANOVA. **B**. Representative image of GFP (green) in the lesion core 14 days after PT (n = 2 mice). Scale bar, 50 µm. **C**. Representative images of Ki67 (red) and GFP (green) in the lesion core 1 and 3 days after PT, compared to uninjured mice. White arrowheads indicate Ki67+GFP+ cells. Scale bar, 20 µm. Quantification of the percentage of Ki67+GFP+ cells normalized by total GFP+ cells (n = 3 mice, uninjured and PT D1; n = 6 mice, PT D3 and PT D6; n = 2 mice, PT D14). *P<0.05 by one-way ANOVA. **D**. Representative images of ApopTag (red) and GFP (green) in the lesion core 1 and 3 days after PT compared to uninjured mice. Scale bars, 20 µm (overviews), 5 µm (magnifications). Quantification of the percentage of ApopTag+GFP+ cells normalized by total GFP+ cells (n = 3 mice, uninjured and PT D1; n = 6 mice, PT D3). **P<0.01 by one-way ANOVA. All data are shown as mean ± s.e.m.

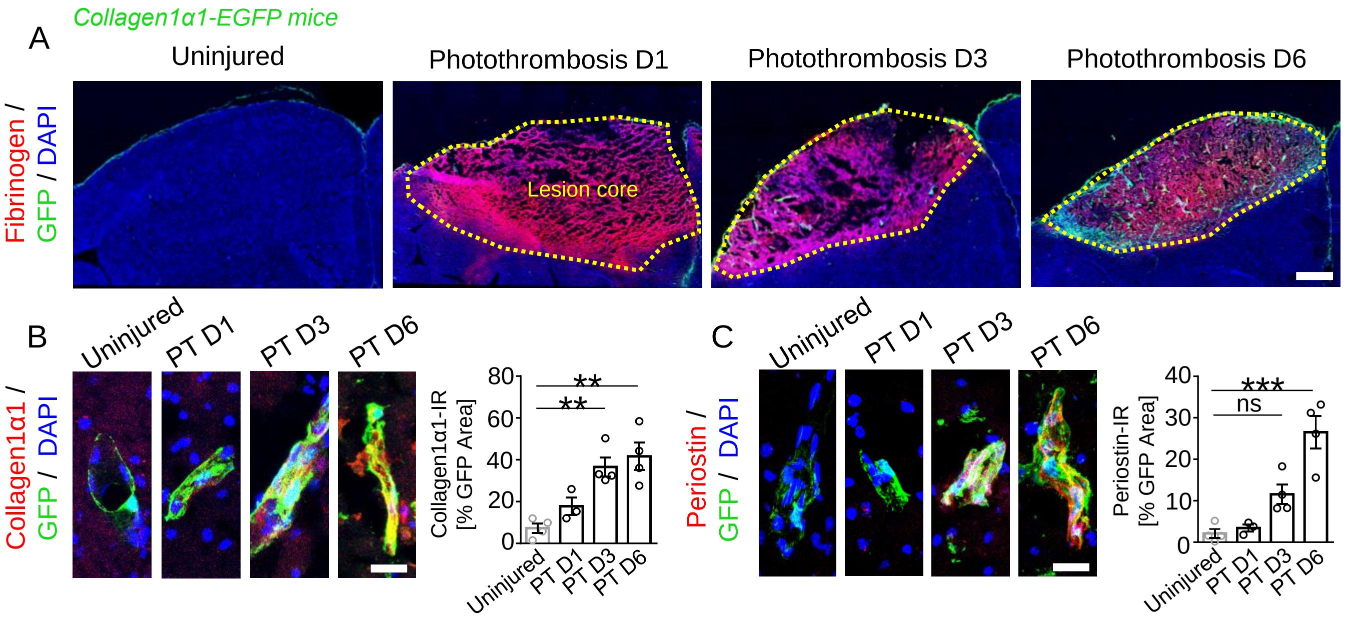

Figure S2 | Fibrinogen deposition in the lesion core and PVF activation in the perivascular space after PT, related to Figure 1.

A. Representative images of fibrinogen (red) and GFP (green) in the lesion core of mice after PT, compared to uninjured brain (n = 2 mice). Scale bar, 400 µm. **B-C**. Representative images of collagen1*α*1 (**B,** red) and periostin (**C,** red) in combination with GFP (green) in the perivascular space in the lesion core of mice after PT, compared to uninjured mice. Scale bars, 20 µm. Quantification of collagen1α1- and periostin–IR per GFP+ area (n = 4 mice, uninjured; n = 3 mice, PT D1; n = 4 mice, PT D3; n = 4 mice, PT D6). ns, not significant, **P<0.01, ***P<0.001 by one-way ANOVA. All data are shown as mean ± s.e.m.

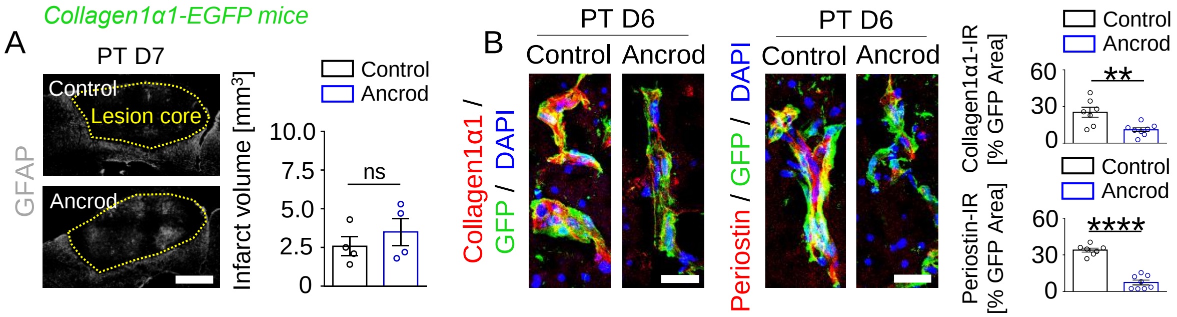

Figure S3 | Fibrinogen depletion effect on lesion size and ECM deposition after PT, related to Figure 2.

A. Immunolabeling of GFAP (grey) in the brain cortex 7 days after PT in ancrod-treated and control mice. The area delineated by the dotted line corresponds to lesion core. Scale bar, 400 µm. Quantification of the infarct volume (n = 4 mice). ns, not significant. **B.** Representative images of Collagen1α1 (red, left) and periostin (red, right) in combination with GFP (green) in the perivascular space in the lesion core of fibrinogen–depleted and control mice 6 days after PT. Scale bars, 20 µm. Quantification of Collagen1α1– and periostin–IR per GFP+ area (n = 7 mice, control; n = 8 mice, ancrod). **P<0.01, ****P<0.0001 by Student´s t test. All data are shown as mean ± s.e.m.

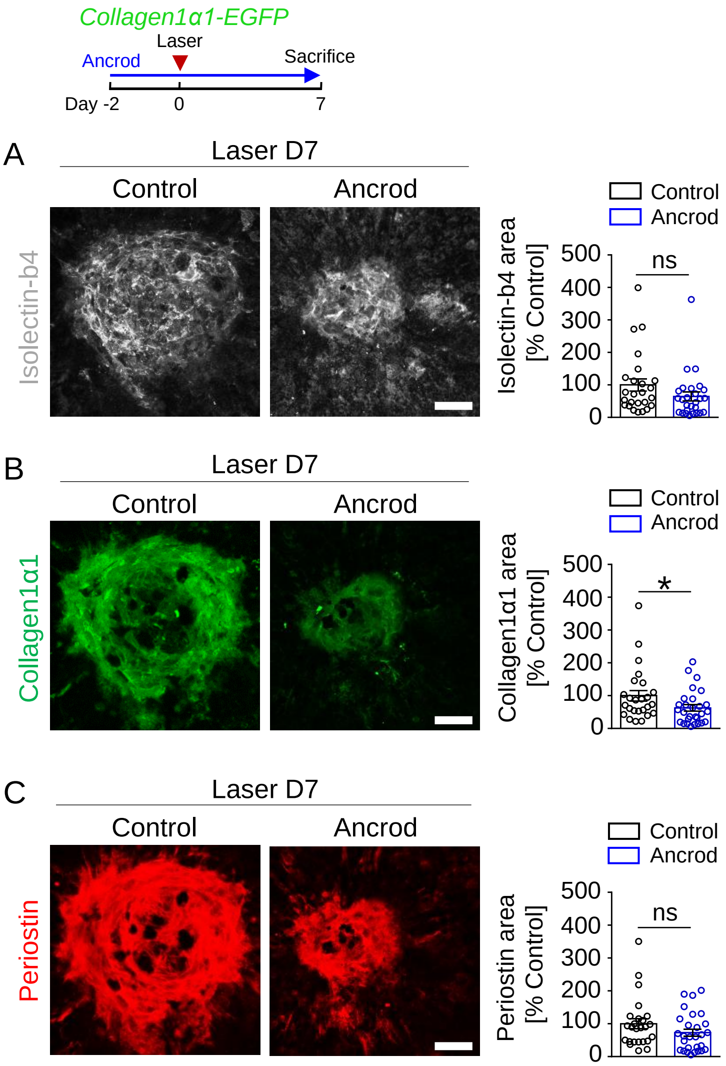

Figure S4 | Fibrinogen depletion reduces ECM deposition in the CNV model, related to Figure 2. **A**. Scheme illustrating laser-induced CNV model in fibrinogen-depleted (ancrod) and control mice. Representative images of isolectin-b4 (grey) in the choroid of fibrinogen-depleted (ancrod) and control mice 7 days after laser. Scale bar, 50 µm. Quantification of isolectin-b4 area in the injured areas of the choroid normalized by the control group (n = 6 mice, n = 25 injured areas in control, n = 27, ancrod). ns, not significant by Mann-Whitney U test. **B**. Representative images of collagen1α1 (green) in the choroid of fibrinogen-depleted (ancrod) and control mice 7 days after laser. Scale bar, 50 µm. Quantification of collagen1α1 area in the choroid normalized by the control group (n = 6 mice, n = 25 injured areas in control, n = 28, ancrod). *P<0.05 by Mann-Whitney U test. **C**. Representative images of periostin (red) in the choroid of fibrinogen-depleted (ancrod) and control mice 7 days after laser. Scale bar, 50 µm. Quantification of periostin area in the choroid normalized by the control group (n = 6 mice, n = 25 injured areas in control, n = 28, ancrod). ns, not significant by Mann-Whitney U test. All data are shown as mean ± s.e.m.

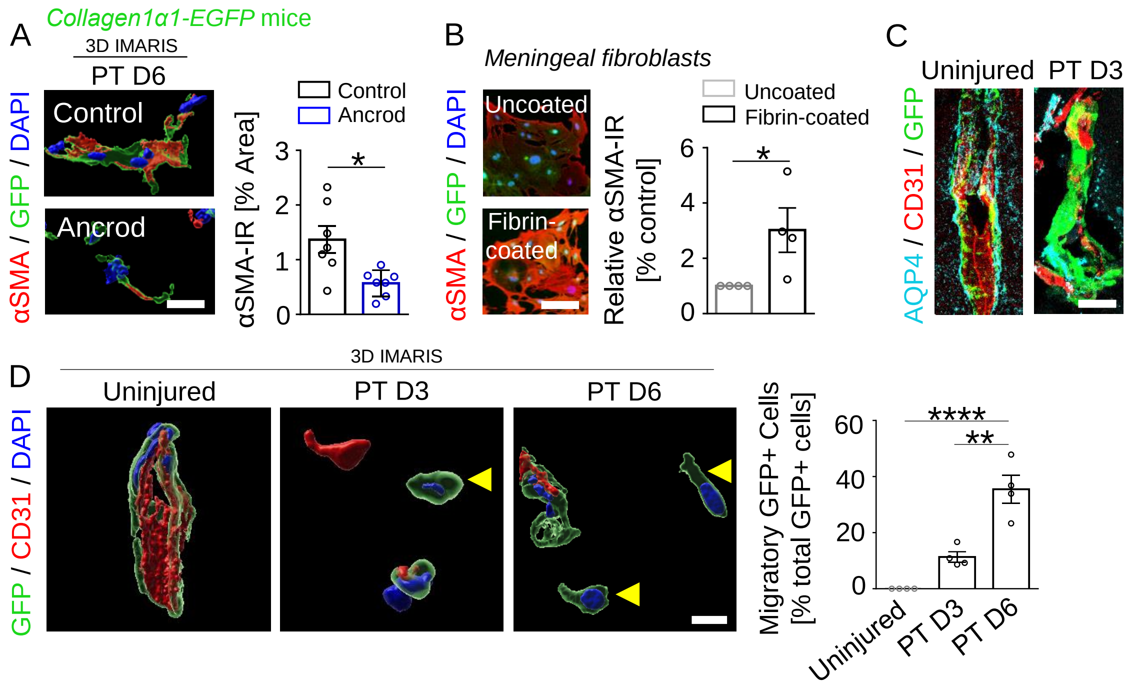

Figure S5 | Fibrinogen-induced αSMA expression in fibroblasts *in vitro*, related to Figure 2.
A. 3D reconstruction of αSMA (red) and GFP (green) in the lesion core of fibrinogen-depleted (ancrod) and control mice 6 days after PT. Scale bar, 5 µm. Quantification of αSMA-immunoreactivity (IR) in the lesion core (n = 7 mice). *P<0.05 by Student's t test. **B**. Representative images of αSMA (red) and GFP (green) in meningeal fibroblasts 2 days after culture on fibrin coating. Scale bar, 5 µm. Quantification of αSMA-IR per cell normalized by the control group (n = 4 biological replicates). *P<0.05 Mann-Whitney U test. **C**. Representative images of AQP4 (cyan), CD31 (red) and GFP (green) in the lesion core of mice 3 days after PT, compared to uninjured mice (n = 4 mice). Scale bar, 10 µm. **D**. 3D reconstruction of GFP (green) and CD31 (red) in the lesion core of fibrinogen–depleted and control mice 3 and 6 days after PT, compared to uninjured mice. Yellow arrowhead indicate migratory PVFs. Scale bar, 20 µm. Quantification of migratory PVFs (n = 4 mice). **P<0.01, ****P<0.0001, by one-way ANOVA. All data are shown as mean ± s.e.m.

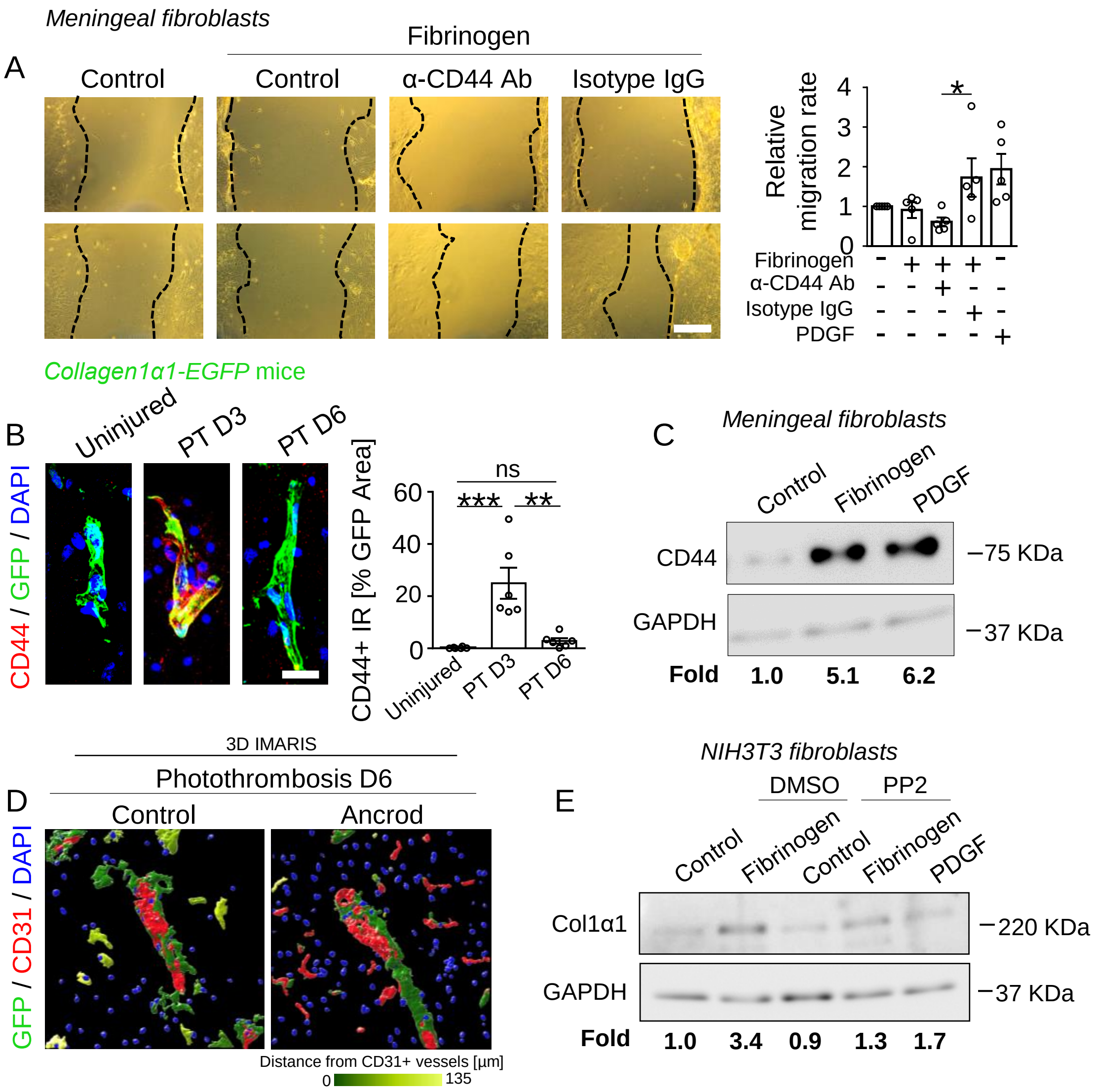

Figure S6 | Fibrinogen-induced CD44 expression in fibroblasts *in vitro*, related to Figure 2.
A. Representative images of meningeal fibroblasts after pretreatment with anti-CD44 blocking antibody or isotype immunoglobulin control for 12 h, followed by fibrinogen treatment for 24 h. PDGF served as a positive control. The scratch was performed after fibrinogen treatment. The extent of scratch closure is delineated by black dashed lines. Scale bar, 50 µm. Quantification of cell migration 24 h after scratch (n = 5 biological replicates). *P<0.05 Mann-Whitney U test. **B**. Representative images of CD44 (red) and GFP (green) in the lesion core of mice 3 and 6 days after PT, compared to uninjured mice. Scale bar, 10 µm. Quantification of CD44-IR per GFP+ area (n = 6 mice). ns = not significant, **P<0.01, ***P<0.001 by one-way ANOVA. **C**. Representative western blot image and quantification of CD44 expressed by meningeal fibroblasts treated for 1 day with fibrinogen. PDGF served as a positive control (n = 5 biological replicates). **D**. 3D reconstruction of GFP (green) and CD31 (red) in the lesion core of fibrinogen-depleted (ancrod) and control mice 6 days after PT. A color scale is set to visually differentiate migratory PVF (lighter green) from PVF attached to the vasculature (darker green) (n = 8 mice). Scale bar, 50 µm. **E**. Representative western blot image and quantification of Collagen1α1 expressed by NIH3T3 cells pretreated for 1 hour with PP2 and treated for 1 day with fibrinogen. PDGF served as a positive control (n = 1 biological replicate). All data are shown as mean ± s.e.m.

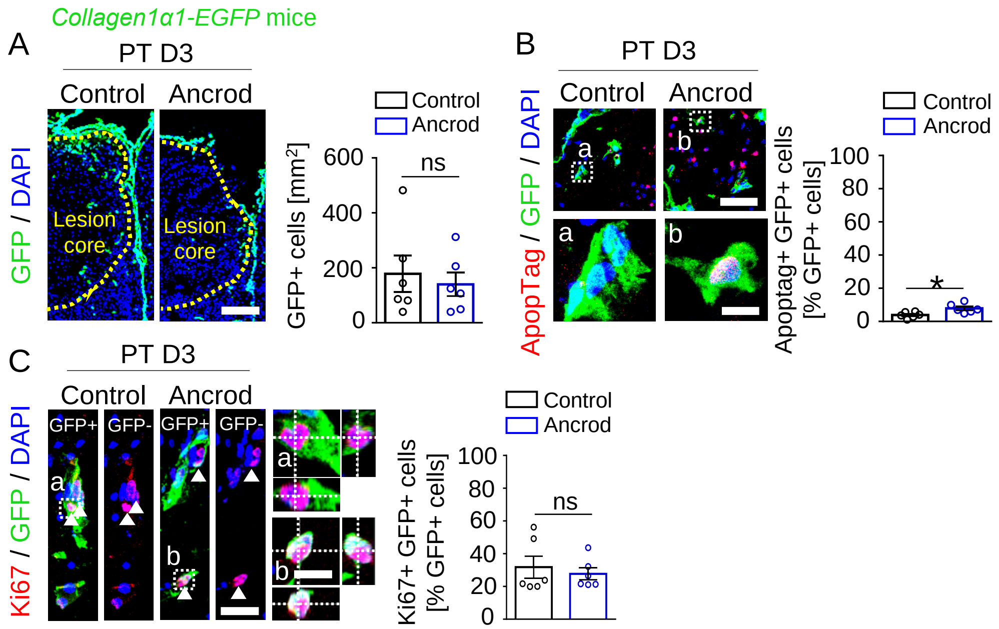

Figure S7 | Fibrinogen depletion effects on PVF cell number, proliferation and apoptosis at day 3 after PT, related to Figure 2.

A. Representative images of GFP (green) in the lesion core of fibrinogen-depleted (ancrod) and control mice 3 days after PT. Scale bar, 150 µm. Quantification of GFP+ cells per area (n = 6 mice). ns, not significant by Mann-Whitney U test. **B**. Representative images of ApopTag (red) and GFP (green) in fibrinogen-depleted (ancrod) and control mice 3 days after PT. Scale bar, 50 µm (overviews), 5 µm (magnifications). Quantification of the percentage of ApopTag+GFP+ cells normalized by total GFP+ cells (n = 6 mice). *P<0.05 by Mann-Whitney U test. **C**. Representative images of Ki67 (red) and GFP (green) and ortogonal views of selected Ki67+GFP+ cells in the lesion core of fibrinogen-depleted (ancrod) and control mice 3 days after PT. White arrowheads indicate Ki67+GFP+ cells. Scale bars, 10 µm (overviews), 5 µm (orthogonal views). Quantification of the percentage of Ki67+GFP+ cells normalized by total GFP+ cells (n = 6 mice). ns, not significant by Mann-Whitney U test. All data are shown as mean ± s.e.m.

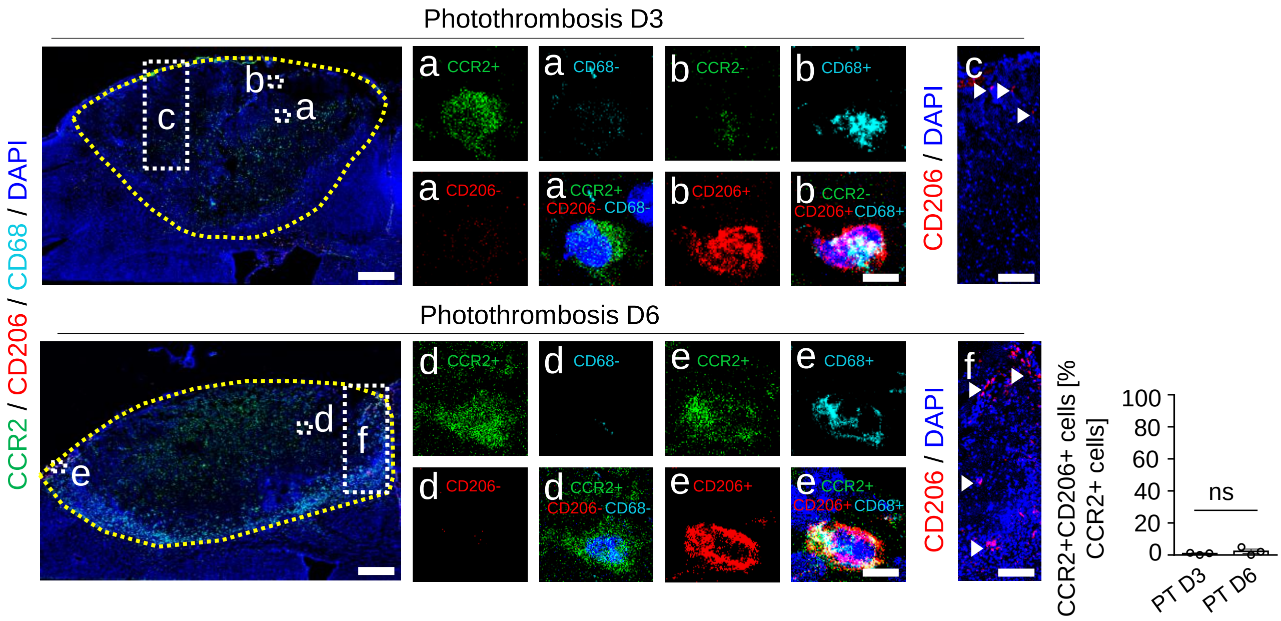

Figure S8 | The majority of CCR2+ cells do not upregulate CD206 expression after PT, related to Figure 3.

Representative image of CCR2 (green), CD206 (red), and CD68 (cyan) in the lesion core 3 days (above) and 6 days (below) after PT. White dashed boxes indicate a CCR2+CD206-CD68- cell (a, d), a CCR2-CD206+CD68+ cell (b) and a CCR2+CD206+CD68+ cell (e) displaying split channels, as well as an enlargement of regions of interest containing CD206+ cells (c, f) (n = 1 mouse, PT D3; n = 3 mice, PT D6). Scale bars, 400 µm (overview), 5 µm (single cells), 100 µm (enlargement). Quantification of CCR2+CD206+ cells normalized to total CCR2+ cells in the lesion core (n = 3 mice). ns, not significant by Mann-Whitney U test. All data are shown as mean ± s.e.m.

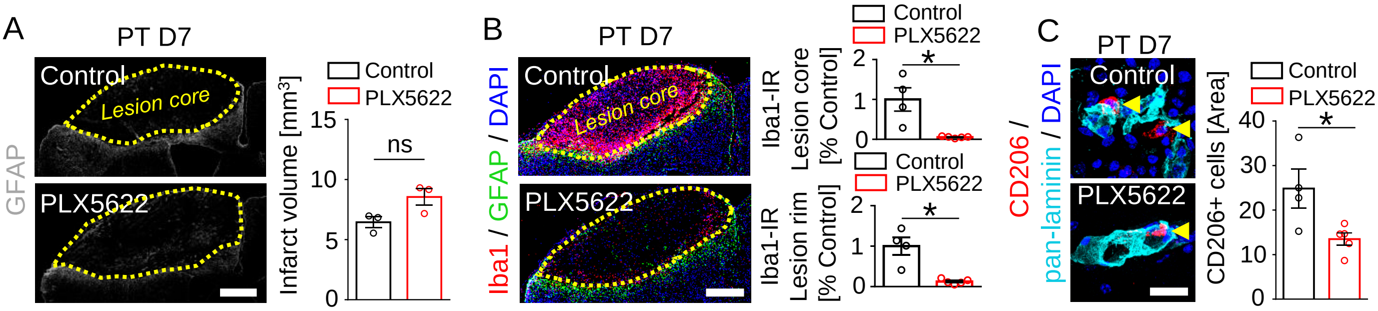

Figure S9 | Myeloid cell depletion effects on lesion size and ECM expression by PVFs, related to Figure 3.

A. Immunolabeling for GFAP (grey) in the brain cortex 7 days after PT in myeloid cell-depleted mice compared to control mice. Scale bar, 400 µm. Quantification of the infarct volume (n = 3 mice). ns, not significant. Unpaired Mann-Whitney test. **B**. Representative images of Iba1 (red) and GFAP (green) in the lesion area in myeloid cell-depleted (PLX5622) mice, compared to control mice 7 days after PT. Scale bar, 400 µm. Quantification of Iba1-IR per lesion core area or lesion rim area normalized by the control group (n = 4 mice, control; n = 5 mice, PLX5622). *P<0.05 by Mann-Whitney U test. **C**. Representative images of CD206 (red) and pan-laminin (cyan) in the perivascular space in the lesion core in myeloid cell-depleted (PLX5622) mice, compared to control animals 7 days after PT. Yellow arrowheads indicate CD206+ perivascular macrophages. Scale bar, 20 µm. Quantification of CD206+ cells per area (n = 4 mice, control; n = 5 mice, PLX5622). *P<0.05 by Mann-Whitney U test. All data are shown as mean ± s.e.m.

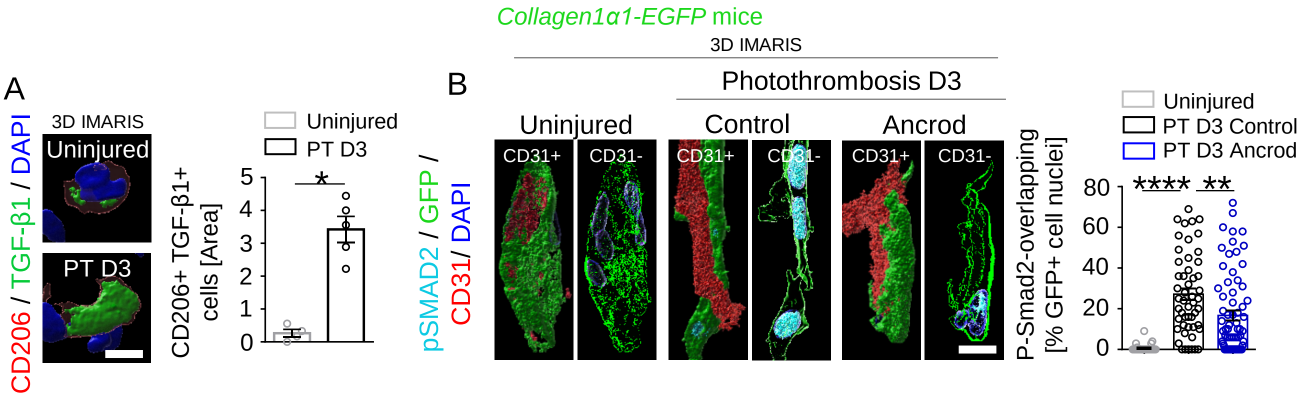

Figure S10 | Early perivascular macrophage activation after PT, related to Figure 3.

A. 3D reconstruction of CD206 (red) and TGF-β1 (green) in the perivascular space in the lesion core 3 days after PT, compared to uninjured mice. Scale bar, 5 µm. Quantification of CD206+ TGF-β1+ cells per area (n = 4 mice, uninjured; n = 5 mice, PT D3). *P<0.05 by Mann-Whitney U test. **B**. 3D reconstruction of pSMAD2 (cyan), GFP (green) and CD31 (red) in the lesion core of fibrinogen-depleted (ancrod) and control mice 3 days after PT, compared to uninjured mice. Scale bar, 20 µm. Quantification of the percentage of overlapping between pSMAD2 and GFP+ nuclei (n = 2 mice; 31 cells, uninjured; 51 cells, PT D3 control; 71 cells PT D3 ancrod). **P<0.01, ****P<0.0001 by one-way ANOVA. All data are shown as mean ± s.e.m.

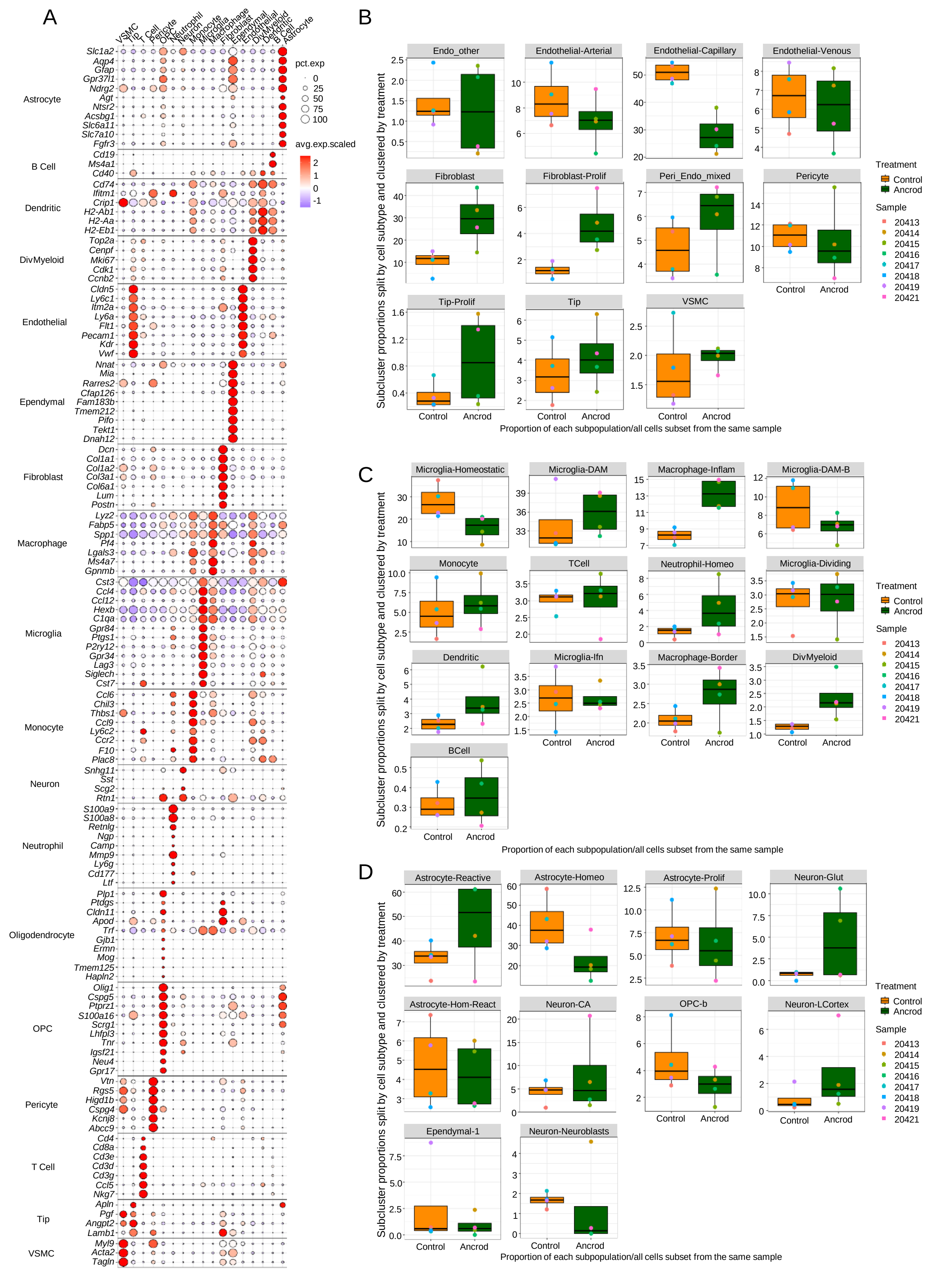
A
VSMC
Tip
T Cell
Pericyte
OPC
Neutrophil
Neuron
Monocyte
Microglia
Macrophage
Fibroblast
Ependymal
Endothelial
DivMyeloid
Dendritic
B Cell
Astrocyte
pct.exp
0
25
50
75
100
avg.exp.scaled
2
1
0
-1
Astrocyte
Slc1a2
Aqp4
Gfap
Gpr37l1
Ndrg2
Agt
Ntsr2
Acsbg1
Slc6a11
Slc7a10
Fgfr3
B Cell
Cd19
Ms4a1
Cd40
Dendritic
Cd74
Ifitm1
Crip1
H2-Ab1
H2-Aa
H2-Eb1
DivMyeloid
Top2a
Cenpf
Mki67
Cdk1
Ccnb2
Endothelial
Cldn5
Ly6c1
Itm2a
Ly6a
Flt1
Pecam1
Kdr
Vwf
Ependymal
Nnat
Mia
Rarres2
Cfap126
Fam183b
Tmem212
Pifo
Tekt1
Dnah12
Fibroblast
Dcn
Col1a1
Col1a2
Col3a1
Col6a1
Lum
Postn
Macrophage
Lyz2
Fabp5
Spp1
Pf4
Lgals3
Ms4a7
Gpnmb
Microglia
Cst3
Ccl4
Ccl12
Hexb
C1qa
Gpr84
Ptgs1
P2ry12
Gpr34
Lag3
Siglech
Cst7
Monocyte
Ccl6
Chil3
Thbs1
Ccl9
Ly6c2
Ccr2
F10
Plac8
Neuron
Snhg11
Sst
Scg2
Rtn1
Neutrophil
S100a9
S100a8
Retnlg
Ngp
Camp
Mmp9
Ly6g
Cd177
Ltf
Oligodendrocyte
Plp1
Ptgds
Cldn11
Apod
Trf
Gjb1
Ermn
Mog
Tmem125
Hapln2
OPC
Olig1
Cspg5
Ptprz1
S100a16
Scrg1
Lhfpl3
Tnr
Igsf21
Neu4
Gpr17
Pericyte
Vtn
Rgs5
Higd1b
Cspg4
Kcnj8
Abcc9
T Cell
Cd4
Cd8a
Cd3e
Cd3d
Cd3g
Ccl5
Nkg7
Tip
Apln
Pgf
Angpt2
Lamb1
VSMC
Myl9
Acta2
Tagln
B
Endo_other
Endothelial-Arterial
Endothelial-Capillary
Endothelial-Venous
Fibroblast
Fibroblast-Prolif
Peri_Endo_mixed
Pericyte
Tip-Prolif
Tip
VSMC
Subcluster proportions split by cell subtype and clustered by treatment
Control
Ancrod
Proportion of each subpopulation/all cells subset from the same sample
Treatment
Control
Ancrod
Sample
20413
20414
20415
20416
20417
20418
20419
20421
C
Microglia-Homeostatic
Microglia-DAM
Macrophage-Inflam
Microglia-DAM-B
Monocyte
TCell
Neutrophil-Homeo
Microglia-Dividing
Dendritic
Microglia-Ifn
Macrophage-Border
DivMyeloid
BCell
Subcluster proportions split by cell subtype and clustered by treatment
Control
Ancrod
Proportion of each subpopulation/all cells subset from the same sample
Treatment
Control
Ancrod
Sample
20413
20414
20415
20416
20417
20418
20419
20421
D
Astrocyte-Reactive
Astrocyte-Homeo
Astrocyte-Prolif
Neuron-Glut
Astrocyte-Hom-React
Neuron-CA
OPC-b
Neuron-LCortex
Ependymal-1
Neuron-Neuroblasts
Subcluster proportions split by cell subtype and clustered by treatment
Control
Ancrod
Proportion of each subpopulation/all cells subset from the same sample
Treatment
Control
Ancrod
Sample
20413
20414
20415
20416
20417
20418
20419
20421

Figure S11 | Cluster identification for the scRNA-Seq dataset and subtype proportions, related to Figure 3.

A. Dot plot of key marker genes used to annotate each cell cluster. Spot size represents the percent of cells in each cluster that express at least one count of the gene. Spot color represents the z-score of expression levels of each gene per-cluster. **B-D**. Bar plots of vascular (B), immune (C), and glial (D) cell subtype proportions-per sample. Proportions are relative to all cells of the same sample for each group. Spots colored by sample and bar plots are colored by treatment.

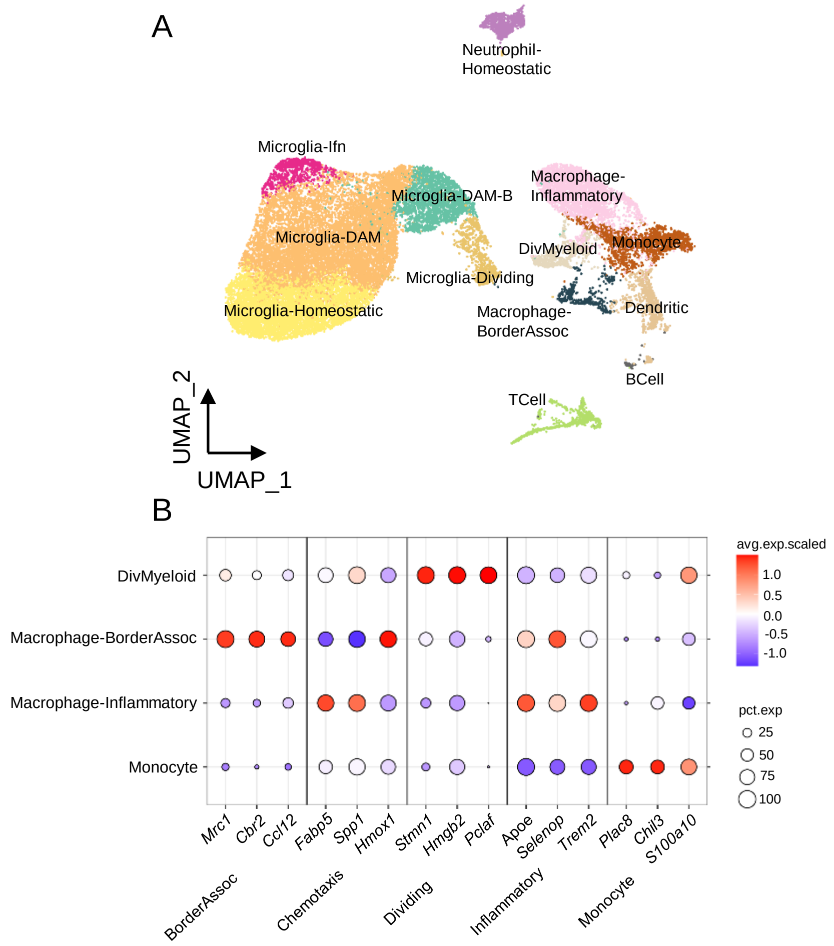

Figure S12 | scRNA-Seq immune cell subcluster identification, related to Figure 3.
A. UMAP representation of distinct immune cell subclusters obtained after subcluster analysis of the identified immune cell cluster in Fig. 3F. **B**. Dot plot of key marker genes used to annotate each cell subcluster. Spot size represents the percent of cells in each subcluster that express at least one count of the gene. Spot color represents the z-score of expression levels of each gene per-subcluster.

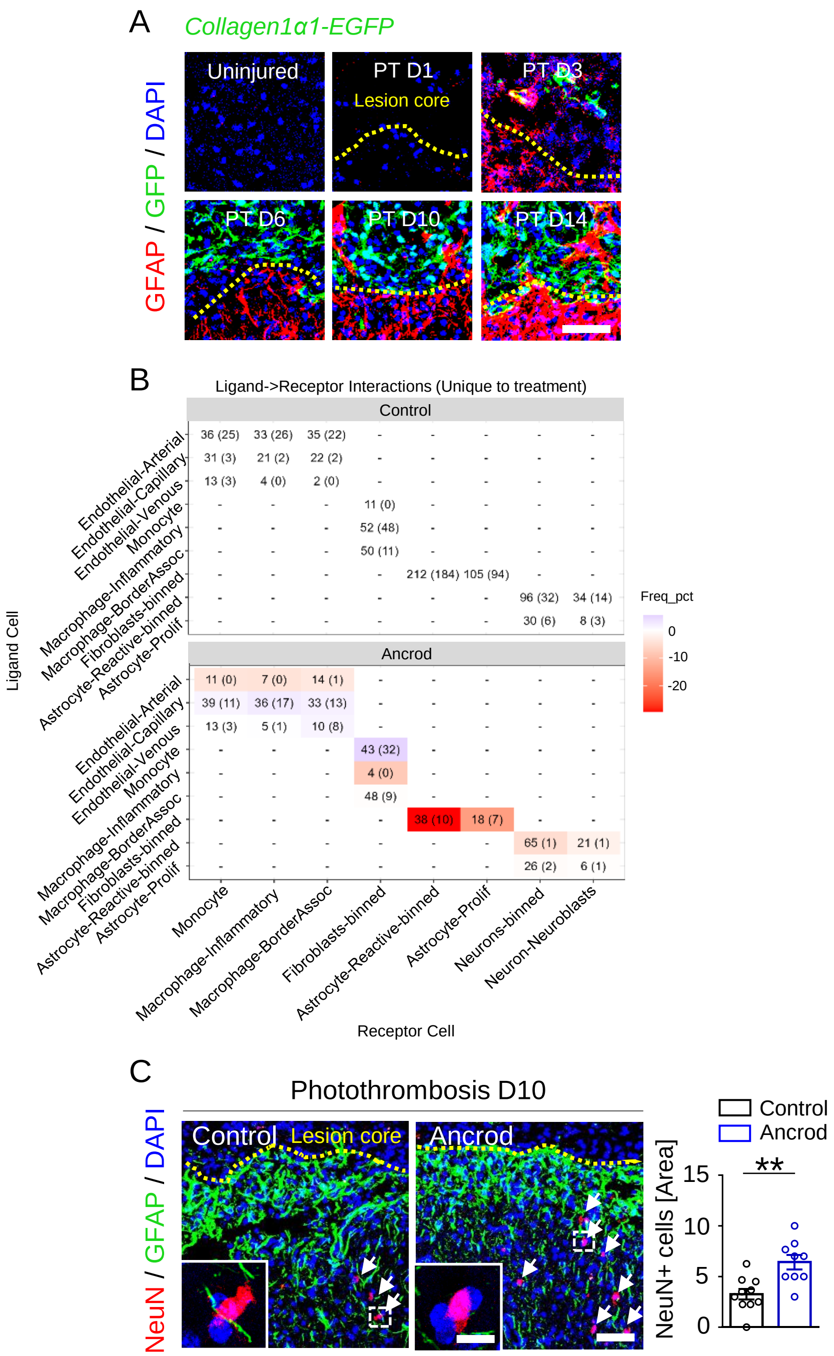

Figure S13 | Fibrinogen depletion reduces PVF-astrocyte cell-cell interaction and improved neuronal survival at the lesion border after PT, related to Figure 4.

A. Representative images of GFP (green) and GFAP (red) at the lesion border in uninjured mice and at different timepoints after PT (n = 1 mouse). Scale bar, 50 μm. **B.** Table of unique ligand-receptor interactions in control- and fibrinogen depleted mice. Ligand cells (y-axis) and receptor cells (x-axis) interactions were filtered for an aggregate rank of <= 0.001 and grouped by treatment. Interactions that were unique to each treatment (n=1 or log fold-change difference of >= 2) are in parentheses. Interactions are colored by percent change in number of interactions from saline to Ancrod (Freq_pct). **C.** Representative images of NeuN (red) and GFAP (green) in the lesion penumbra of fibrinogen-depleted (ancrod) and control mice 10 days after PT. White arrows indicate NeuN+ cells. Scale bars, 50 μm (overviews), 10 μm (magnifications). Quantification of NeuN+ cells in the lesion penumbra (n = 10 mice, control; n = 9 mice, ancrod). **P<0.01 by Student's t test. All data are shown as mean ± s.e.m.

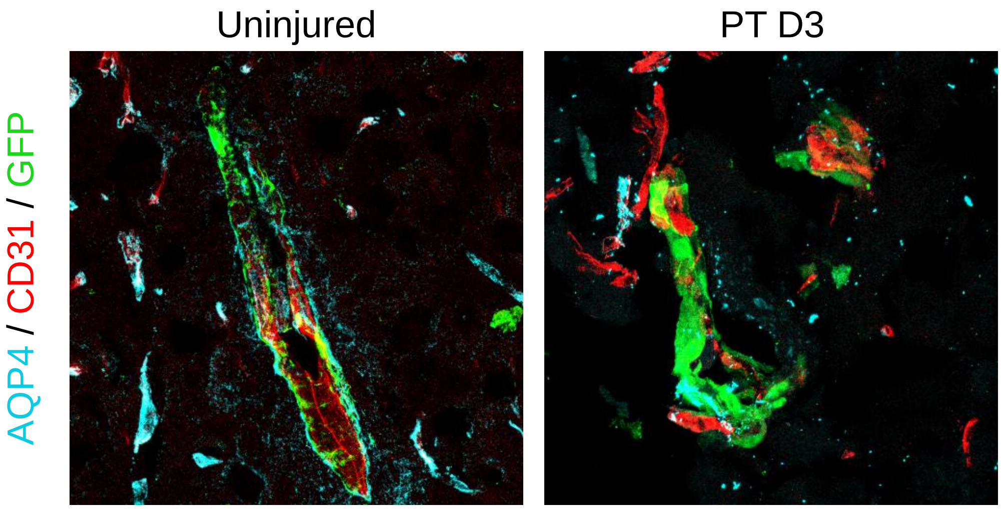

Data S1 | Representative images of AQP4 (cyan), CD31 (red) and GFP (green) in the lesion core of mice 3 days after PT, compared to uninjured mice (n = 4 mice).
